# Parallel Metabolomic Profiling of Cerebrospinal Fluid and Serum for Identifying Biomarkers of Injury Severity after Acute Human Spinal Cord Injury

**DOI:** 10.1038/srep38718

**Published:** 2016-12-14

**Authors:** Yiman Wu, Femke Streijger, Yining Wang, Guohui Lin, Sean Christie, Jean-Marc Mac-Thiong, Stefan Parent, Christopher S. Bailey, Scott Paquette, Michael C. Boyd, Tamir Ailon, John Street, Charles G. Fisher, Marcel F. Dvorak, Brian K. Kwon, Liang Li

**Affiliations:** 1 Department of Chemistry, University of Alberta, Edmonton, AB, T6G2G2, Canada; 2 International Collaboration on Repair Discoveries (ICORD), University of British Columbia, Blusson Spinal Cord Centre, 818 West 10^th^ Avenue, Vancouver, BC, V5Z 1M9, Canada; 3 Department of Computing Science, University of Alberta, Edmonton, AB, T6T 2E8, Canada; 4 Division of Neurosurgery, Dalhousie University, Halifax Infirmary, 1796 Summer Street, Halifax, NS, B3H 3A7, Canada; 5 Hôpital du Sacré-Coeur de Montréal, 5400 Boul Gouin O, Montréal, QC, H4J 1C5, Canada; 6 Chu Sainte-Justine, Dept. of Surgery, Université de Montréal, PO Box 6128, Station Centre-ville, Montreal, QC, H3C 3J7, Canada; 7 Division of Orthopaedic Surgery, Schulich Medicine & Dentistry, Victoria Hospital 800 Commissioners Road East, Room E4 120, London, ON, N6C 5W9, Canada; 8 Division of Neurosurgery, University of British Columbia, Vancouver Spine Surgery Institute, 818 West 10^th^ Avenue, Vancouver, BC, V5Z 1M9, Canada; 9 Department of Orthopaedics, University of British Columbia, Vancouver Spine Surgery Institute, 818 West 10^th^ Avenue, Vancouver, BC, V5Z 1M9, Canada

## Abstract

Suffering an acute spinal cord injury (SCI) can result in catastrophic physical and emotional loss. Efforts to translate novel therapies in acute clinical trials are impeded by the SCI community’s singular dependence upon functional outcome measures. Therefore, a compelling rationale exists to establish neurochemical biomarkers for the objective classification of injury severity. In this study, CSF and serum samples were obtained at 3 time points (~24, 48, and 72 hours post-injury) from 30 acute SCI patients (10 AIS A, 12 AIS B, and 8 AIS C). A differential chemical isotope labeling liquid chromatography mass spectrometry (CIL LC-MS) with a universal metabolome standard (UMS) was applied to the metabolomic profiling of these samples. This method provided enhanced detection of the amine- and phenol-containing submetabolome. Metabolic pathway analysis revealed dysregulations in arginine-proline metabolism following SCI. Six CSF metabolites were identified as potential biomarkers of baseline injury severity, and good classification performance (AUC > 0.869) was achieved by using combinations of these metabolites in pair-wise comparisons of AIS A, B and C patients. Using the UMS strategy, the current data set can be expanded to a larger cohort for biomarker validation, as well as discovering biomarkers for predicting neurologic outcome.

Acute spinal cord injury (SCI) often causes paralysis and severe disability, for which there are few treatments that even marginally improve neurologic outcome. In Canada, the incidence of acute traumatic SCI is over 1,500 per year^1^, and the estimated annual economic impact of SCI is over $2 billion^2^. As such, SCI has emerged as a major public health issue in modern society and has prompted considerable efforts to develop therapeutic strategies to improve neurologic outcome and reduce the burden of disability. Many of these efforts have focused on the acutely injured patient, as it is expected that interventions applied at this early stage of injury afford the opportunity to reduce secondary injury and, through such ‘neuroprotection’, promote improved outcome.

Efforts to validate potential interventions in the acute SCI setting have been hampered by the reliance upon clinical assessments to quantify neurologic impairment after SCI and to stratify the severity of injury. These assessments are imprecise predictors of neurologic recovery and are often impossible to even obtain in the acute setting due to intoxication/sedation or concomitant injuries^3^. Biomarkers that objectively characterize injury severity and more precisely predict neurologic recovery would be extremely helpful in facilitating the evaluation of novel treatments in acute SCI clinical trials.

Additionally, the scientific development of novel therapies for acute SCI depends upon an understanding of the complex pathophysiologic mechanisms that are triggered after acute SCI. Traumatic injury to the spinal cord induces multiple disturbances in the human metabolic network, including oxidative stress, glycolysis, and amino acid and lipid metabolism^4^,^5^,^6^,^7^. Metabolomics is an emerging field for high-throughput global profiling of the collection of all metabolites in a biological system (i.e., the metabolome). Such profiling may also identify metabolite candidates that can be utilized as potential biomarkers. While almost all of our understanding of the metabolite changes after SCI has come from small animal models of SCI^8^,^9^,^10^,^11^, a global profiling of the metabolic network in response to acute *human* SCI has not been previously reported.

In this study, we describe a parallel metabolomic profiling of cerebrospinal fluid (CSF) and serum from acute SCI patients using a newly developed chemical isotope labeling liquid chromatography mass spectrometry (CIL LC-MS) platform with a universal metabolome standard (UMS)^12^. Our objectives were to characterize the metabolite changes that occur in these two biofluids in response to injury, and to identify potential biomarkers of injury severity. We utilized dansylation LC-MS to enable relative quantification of the amine- and phenol-containing submetabolome with high coverage. With the UMS, the data set reported herein can be expanded in the future by analyzing additional samples for biomarker validation as well as discovering prognostic biomarkers for predicting neurologic recovery.

## Results

### Clinical characteristics of study subjects

We prospectively enrolled 30 acute traumatic SCI patients who had suffered a cervical or thoracolumbar injury (C3-L1) in whom a valid baseline neurologic examination could be performed in accordance with the International Standards for Neurologic Classification of SCI (ISNCSCI). This clinical examination was done typically within 24 hours of injury. The ISNCSCI examination and its scoring conventions are available from http://asia-spinalinjury.org/wp-content/uploads/2016/02/International_Stds_Diagram_Worksheet.pdf and this form is also available from the corresponding authors upon request. The baseline American Spinal Injury Association (ASIA) Impairment Scale (AIS) grade was A for 10 patients, B for 12, and C for 8. In general terms, AIS A denotes those with complete motor and sensory paralysis (the most severe neurologic impairment), AIS B denotes those with complete motor paralysis but some preserved sensation, and AIS C is assigned when there is some preserved motor and sensory function. A lumbar intrathecal catheter was inserted at the time of surgery and maintained for 3–5 days post-injury for the acquisition of serial CSF samples. CSF and serum samples were obtained at 3 time points: approximately 24, 48, and 72 hours post-injury. The CSF and blood samples were drawn within a few minutes of one another and were processed, centrifuged, and frozen at the bedside. The patients were examined clinically at 6 months post-injury to determine AIS grade and motor score recovery.

### Metabolomic analysis of CSF and serum

**Figure 1:**
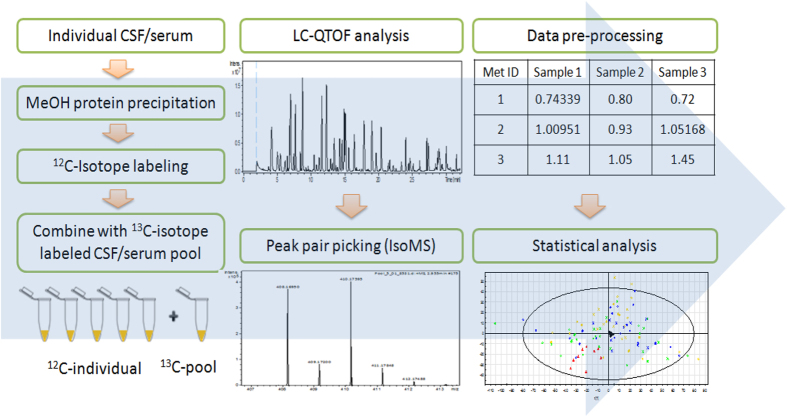
Overall workflow for differential isotope labeling metabolomic profiling of CSF and serum.

Figure 1 shows the overall workflow of CIL LC-MS for profiling the submetabolomes of CSF and serum in this cohort of 30 acute SCI patients. Prior to LC-MS analysis, each ^12^C*-*labeled individual CSF or serum sample was combined with an equal mole amount of the corresponding ^13^C-labeled pooled sample which served as the UMS. The relative concentration of each metabolite in an individual sample to that of the corresponding metabolite in UMS was measured using the intensity ratio of the ^12^C/^13^C peak pair. Since the same UMS was spiked into all the comparative samples, the peak ratio values of a given metabolite in individual samples reflected their concentration differences in these samples. The use of ^13^C-labeled UMS as internal standards enables more accurate quantification of the ^12^C-labeled metabolites. In addition, any future samples could be ^12^C-labeled and then compared to the ^13^C-labeled UMS, thereby allowing expansion of the current dataset to a larger cohort. Dansylation LC-MS has the advantage of improved chromatographic separation and enhanced electrospray ionization (ESI) response, resulting in 10- to 1000-fold increase in detection sensitivity^13^. A detailed discussion of this analytical platform, including the workflow, evaluation of analytical variability and peak detectability, as well as comparison between the CSF and serum submetabolomes is included in Supplemental Note S1. Using this method of targeting the amine/phenol-containing metabolites, we were able to detect 1213 and 2316 ^12^C/^13^C peak pairs from CSF and serum, respectively. By matching accurate mass and retention time with the dansyl standard library^14^ (mass error <5 ppm and retention time error <30 s) or with authentic standards, we identified 120 peak pairs which belong to 110 metabolites (see Supplemental Table T1). Among these 110 identified metabolites, 100 were common to the two biofluids, while 2 of them could only be detected in CSF and 8 of them were only detectable in serum (see Note in Table T1).

### Metabolomic Profiles in Spinal Cord Injury

**Figure 2:**
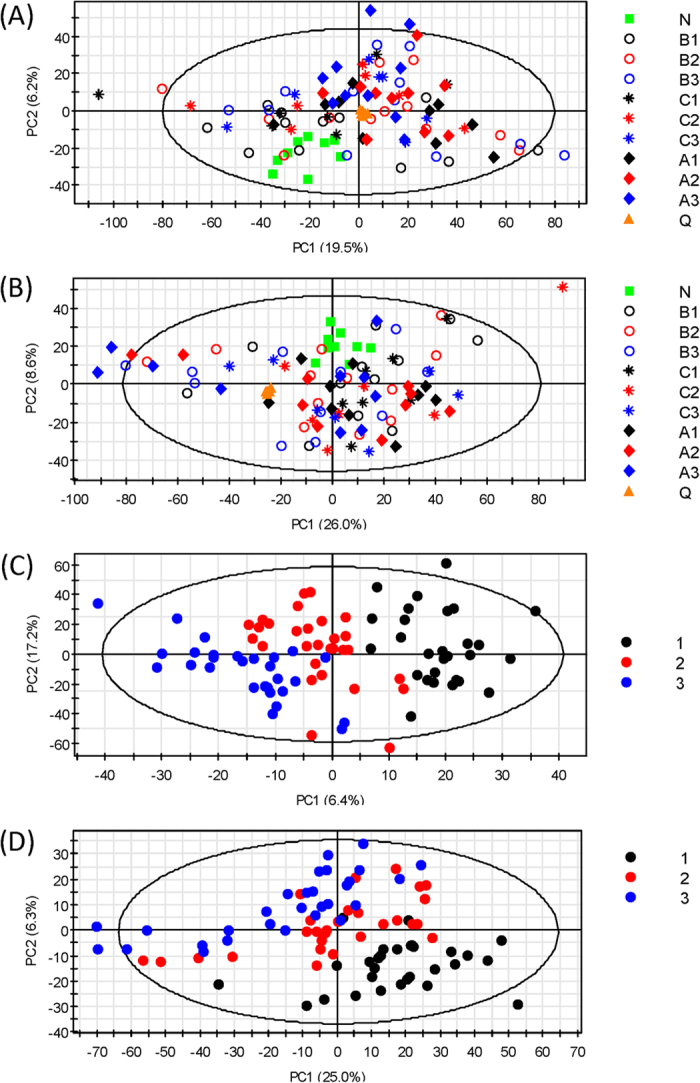
PCA score plots for (**A**) serum and (**B**) CSF. Green box: non-injured control; black: t_1_; red: t_2_; blue: t_3_; diamond: AIS A; circle: AIS B; star: AIS C; orange triangle: quality control. PLS-DA score plots for (**C**) serum and (**D**) CSF. Black: t_1_; red: t_2_; blue: t_3_. The PLS-DA model was cross-validated using twenty permutation tests.

Principle component analysis (PCA) was first applied to provide an overview of the metabolomic dataset. Figure 2A and B show the PCA score plots for all serum and CSF samples analyzed. The six QC samples were clustered close together, indicating good instrument stability throughout the LC-MS analysis. We then applied partial least squares discriminant analysis (PLS-DA) to investigate how metabolites change over time after injury. In this study, the CSF and serum samples were analyzed at three time points approximating 24, 48, and 72 hours: t_1_ (16–32 hours), t_2_ (40–56 hours) and t_3_ (64–80 hours). Figure 2C and D show the PLS-DA score plots and Supplemental Figure S1 shows validation of the PLS-DA models using the 20-permutation test. For both R^2^ and Q^2^ the slopes are positive and the permutation data are lower than the original point, suggesting that the PLS-DA models are valid. A clear separation is observed between samples collected at t_1_ and t_3_, while the t_2_ samples fall in between t_1_ and t_3_ with some overlap. This pattern suggests a gradual change in the metabolic profile from t_1_ to t_3_.

**Figure 3:**
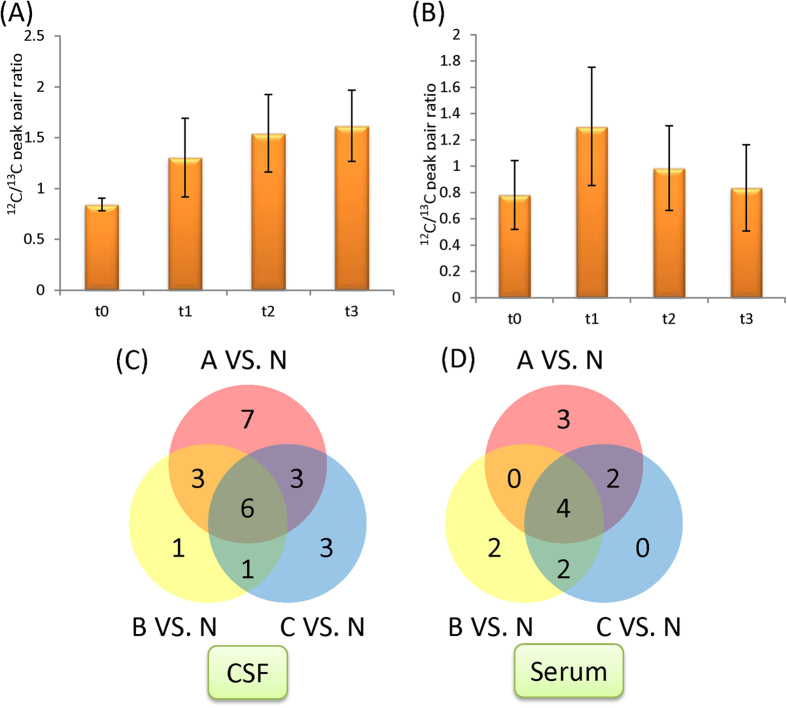
Examples showing how metabolite change with time. (**A**) Pattern 1: gradual increase or decrease over time; (**B**) pattern 2: an abrupt change at t_1_ followed by gradual restoring of the metabolite level. Venn diagram showing the number of metabolites that are significantly different (fold change >1.5, p < 0.05) between injured and non-injured samples in (**C**) CSF and (**D**) serum. A: AIS A; B: AIS B; C: AIS C; N: non-injured control.

With the non-injured controls serving as the reference point (t_0_), we found that the temporal changes follow two major patterns. The first pattern is a gradual increase or decrease of the metabolite level over time (see Fig. 3A), and the second pattern involves an abrupt change during the first 16 to 32 h after injury (i.e., the t_1_ samples), followed by a gradual restoring of the metabolite level back to the non-injured state (see Fig. 3B). Based on the Variable Importance in Projection (VIP) score of greater than 1.5 and *p* < 0.05 in analysis of variance (ANOVA), 376 serum and 73 CSF metabolites that exhibit statistically significant temporal changes were chosen. Among these differentiating metabolites, 20 of them were commonly detected in serum and CSF. The lower number of significant metabolites in CSF is not unexpected as the breadth of the metabolome in CSF is much smaller compared to serum. Among the 376 serum metabolites, 142 (38%) of them follow pattern 1 and 227 (60%) conform to pattern 2. For the 73 CSF metabolites, pattern 1 accounts for 13 (18%) and pattern 2 explains 49 (67%) of the temporal changes. This observation suggests that most of the metabolic changes occur at an early stage of the injury (i.e., pattern 2). Metabolic changes in the first 16 to 32 h after injury are of great interest because they are responses that represent targets for acute pharmacologic/biologic interventions and are also potentially useful for objectively assessing injury severity at an early stage. Therefore, we focused our analysis on the t_1_ samples for the following metabolomics analysis.

### Targeted metabolic profiling of injury-induced changes

In this work, we have positively identified 110 metabolites (Supplemental Table T1) that cover 42 metabolic pathways, which allow us to perform targeted metabolic profiling and evaluation of perturbed pathways triggered by SCI.

We first compared the metabolic profiles of each of the injury groups (A, B and C) with the non-injured controls (N). As the total number of samples was relatively small, we only performed univariate analyses to identify metabolites that were significantly altered. The number of metabolites that met the selection criteria (fold change >1.5, *p* < 0.05) in at least one of the three pairwise comparisons was illustrated by the Venn diagram (see Fig. 3C,D), and Table 1 lists the fold change and *p*-values and *q*-values of these metabolites. Amongst a total of 24 CSF and 13 serum compounds identified, 6 CSF and 4 serum metabolites were detected at significantly different levels in all three injury groups (Table 1). In CSF, these metabolites were uridine, imidazoleacetic acid, methionine sulfoxide, arginine, cystathionine and homocarnosine. In serum, these were uridine, 4-hydroxyproline, N1, N12-diacetylspermine and glycylproline. These commonly detected metabolites are of particular interest to represent the characteristic of the injury state. It was also noted that one endogenous metabolite, uridine, was present at lower levels in both CSF and serum of the SCI patients. In addition, small fold changes were observed in most significant metabolites, likely due to the fact that metabolic network in general maintains a very high homeostasis. However, even small changes of individual metabolites in a particular pathway could indicate that the particular pathway was perturbed by an event such as SCI in this study. Fortunately, our CIL LC-MS technique is sufficiently accurate and precise to detect small fold changes with high confidence.

### Pathway analysis of injury-induced metabolic changes

**Figure 4:**
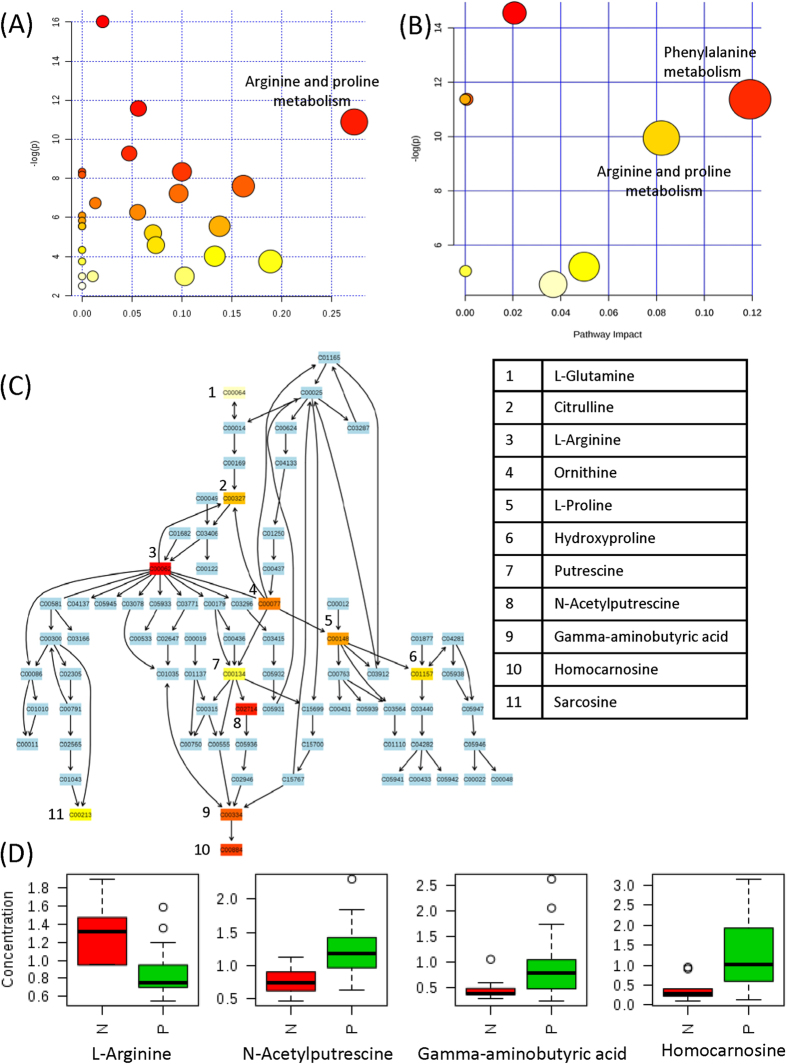
(**A**) Overview of pathway analysis based on selected CSF metabolites. (**B**) Overview of pathway analysis based on selected serum metabolites. Each circle represents a matched pathway. The node color and radius were determined by *p*-value and pathway impact value, respectively. A detailed summary of MetPA results can be found in Supplemental Table T2. (**C**) Schematic illustration of the arginine and proline metabolism. Compounds were represented by their KEGG compound ID. Matched compounds were shown in varied heat map colors based on their *p*-values, with red indicates lower *p*-value. The names of the eleven metabolites detected in this study were listed in the table on the right. The peak ratios of four most significantly altered metabolites in CSF were shown below as box-and-whisker plots (**D**). N: non-injured controls; P: injured patients.

For the 24 CSF and 13 serum metabolites whose levels were significantly different in SCI patients as compared to non-injured controls, a metabolic pathway analysis (MetPA) was performed to evaluate their associated pathways. Figure 4A and B show an overview of the pathway analysis for CSF and serum samples, respectively, with the x-axis representing the pathway impact value calculated from pathway topological analysis and the y-axis corresponding to the −log (*p*) value obtained from pathway enrichment analysis. Supplemental Table T2 provides a summary of the MetPA results. The pathways that are most relevant to the injury response should be characterized by both high −log (*p*) (i.e., low *p*-value) and high pathway impact values. As shown in Fig. 4A, the most significantly affected pathway in CSF, as located on the top-right corner, is arginine and proline metabolism (Fig. 4C). In particular, we observed decreased levels of arginine, and increased levels of downstream metabolites including N-acetylputrescine, gamma-aminobutyric acid (GABA) and homocarnosine (Fig. 4D). For serum, the pathways that are important in both enrichment and topological analysis are phenylalanine metabolism and arginine and proline metabolism. Only one hit, phenylalanine, was found in phenylalanine metabolism. In arginine and proline metabolism, an increase in N-acetylputrescine and decreases in sarcosine and hydroxyproline were observed.

### Targeted metabolic profiling of different injury severities

**Figure 5:**
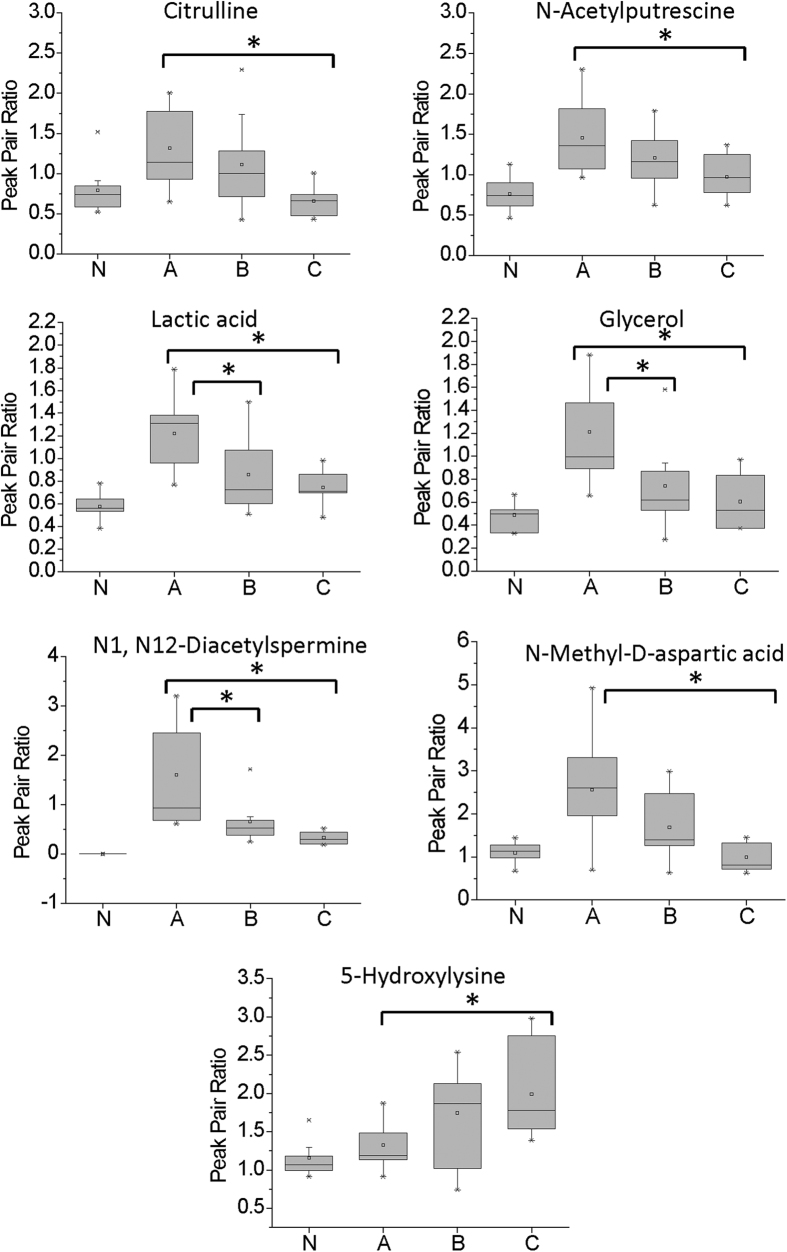
Box-and-whisker plot showing the relative abundance (expressed as peak pair ratios) of selected metabolites in different injury groups (A, B and C) and non-injured controls (N). The asterisk indicates a significant difference among AIS A, B and C (*p* < 0.05).

To find metabolic signatures for evaluating the injury severity, we performed analysis of variance (ANOVA) with Tukey’s *post-hoc* test to select metabolites that had significantly difference within-group and between-group means (*p* < 0.05). It was observed from the PCA score plots (Fig. 2A and B) that the differences between AIS A, B, and C spinal cord injury groups were considerably smaller than between SCI and non-injured samples. As a result, the number of differentiating metabolites among three SCI injury severities (AIS A, B, C) was lower. However, within CSF we identified six metabolites whose levels were influenced by injury-severity, including citrulline, glycerol, lactic acid, N-acetylputrescine, N1, N12-diacetylspermine and N-methyl-D-aspartic acid. For serum, only one metabolite, 5-hydroxylysine, met this criterion. Figure 5 presents the box-and-whisker plots of these metabolites in the three injury groups, as well as in the non-injured controls. It is noted that for all six CSF metabolites, the metabolite levels were the highest in the AIS A group, followed by AIS B, while the non-injured controls were the closest to group C. Metabolites that were significantly different in pairwise comparisons via the *post-hoc* test were labeled with an asterisk in Fig. 5. The metabolite levels were significantly different between A and C for all seven metabolites, and between A and B for three of the metabolites: N1, N12-diacetylspermine, glycerol and lactic acid.

### Classification of different severities of neurologic injury using metabolites

**Figure 6:**
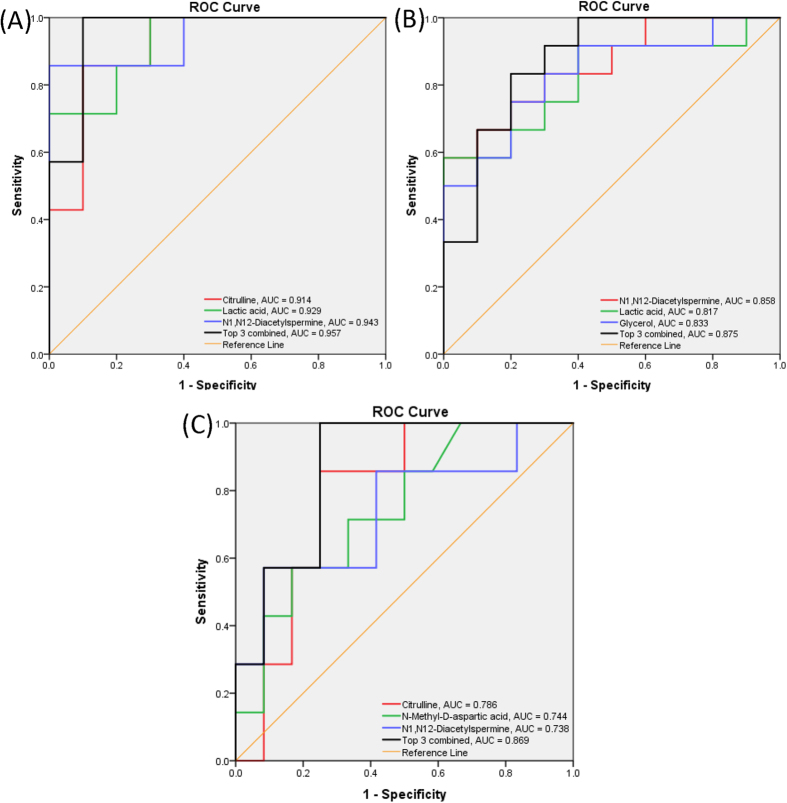
Receiver operating characteristic (ROC) curves summarizing the classification performance of three individual metabolites with highest AUC values, and their combined probability calculated from logistic regression. (**A**) Comparison between AIS A and C; (**B**) comparison between AIS A and B and (**C**) comparison between AIS B and C.

We then examined whether the selected metabolites or combinations thereof could be used to classify AIS A, B and C levels. We first evaluated the receiver-operating characteristic (ROC) curves for each pairwise comparison (i.e., A vs. B, A vs. C and B vs. C). Supplemental Table T3 summarizes the sensitivity, specificity, area under the curve (AUC) and 95% confidence level (95% CI) for each metabolite. We observed large AUC values (AUC ≥ 0.9) between AIS A and C for four metabolites used alone: N1, N12-diacetylspermine, lactic acid, citrulline and glycerol, with good sensitivity and specificity at the cut-off threshold. In addition, by using a combination of multiple metabolites, the AUC value could be further increased. For example, when N1, N12-diacetylspermine, lactic acid and citrulline were used as a panel of biomarkers, the AUC value was increased to 0.957 (Fig. 6A). For comparison between AIS A and AIS B, AUC values of greater than 0.8 were observed for N1, N12-diacetylspermine, lactic acid and glycerol used individually, and a larger AUC value (0.875) was obtained when these metabolites were used together (Fig. 6B). These results suggest that these selected metabolites could be useful for potentially differentiating AIS A from B and C at an early stage of acute SCI. On distinguishing AIS B and C, some of the individual metabolites gave fair (0.7–0.8) AUC values. However, the use of a three-metabolite combination could increase the AUC value to 0.869 (Fig. 6C).

In addition to ROC analysis, we also applied logistic regression to create prediction models in combination with leave-one-out cross validation to evaluate prediction accuracy. The prediction model was built in two steps. Firstly, a logistic regression model was applied for predicting AIS A vs. non-AIS A on 29 samples (one patient sample at t_1_ was missing). Then, the non-AIS A samples identified from step 1 were subjected to a second logistic analysis that predicted AIS C vs. non-AIS C. The samples that were predicted as non-AIS C in the second step were assigned AIS B. A 3 × 3 confusion matrix was then built based on the predicted results. The results are shown in Supplemental Table T4 along with the equations used in the logistic regression model. In this case, the metabolites used to build the two-step models were 1) citrulline, glycerol, N-methyl-D-aspartic acid, and 2) citrulline and glycerol, which were selected using the greedy stepwise approach. This two-step logistic regression model accurately classified the AIS grades in 25 out of 29 cases (86.2%) in the first step and 14 out of 19 cases in the second step (73.7%), with an overall accuracy of 72.4% (i.e., 21 out of 29 cases).

### Non-targeted metabolic analysis for injury level classification

We further investigated whether using a set of non-targeted metabolites (i.e., all the metabolites including those not identified) could build prediction models with improved classification accuracy. All serum and CSF metabolites with ANOVA *p* < 0.05 were extracted, and the greedy stepwise approach was used to rank and select the top metabolites. We applied the same strategy described above to build two-step logistic regression models. Three models were built based on using CSF metabolites only (Supplemental Table T5), using serum metabolites only (Supplemental Table T6), and using a combination of serum and CSF metabolites (Supplemental Table T7). The overall prediction accuracies were 93.1% for both CSF model and combined serum/CSF model, and 86.2% for serum model. Some of these metabolites were putatively identified by matching accurate mass against the MyCompoundID database or the human metabolome database (HMDB), and a complete list of these putative metabolites was given in Supplemental Table T8. Compared to making a prediction with only the identified metabolites (Supplemental Table T4), the use of top-ranked metabolites selected by non-targeted analysis provided significantly improved prediction accuracy, and the best prediction result was achieved using either a panel of CSF metabolites or a panel of combined CSF and serum metabolites.

## Discussion

Recently, it has been shown that metabolomic screening of rat plasma samples can be used to establish an injury severity evaluation model based on the identified metabolomic fingerprints^15^. However, metabolomic analysis of human SCI samples has not been previously reported. CSF is considered to be a more specific and informative biofluid than blood for studying the injured spinal cord and brain because of its proximity and its metabolic simplicity^16^,^17^. Although the metabolic profile in blood has been reported to resemble that in CSF in other neurological diseases^18^,^19^, the relationship between metabolic changes in CSF and blood following SCI remains to be investigated.

While CSF and serum are both promising biofluids for discovery of potential SCI biomarkers, the metabolome analysis of CSF is challenged by the relatively low metabolite concentrations. In this study, we applied a dansylation labeling technique for metabolomic profiling of CSF and serum samples, which provides a signal enhancement of 10- to 1000-fold on its targeted submetabolome. As shown in Fig. 4 of Supplemental Note S1, dansylated metabolites were detected predominantly and distributed evenly over the entire retention time window in LC-MS. Because of the improved ESI response and chromatographic separation, we were able to achieve a more comprehensive analysis of the amine- and phenol-containing submetabolome. In total, 1213 and 2316 peak pairs or metabolites could be detected in CSF and serum, respectively, and 102 CSF and 108 serum metabolites could be positively identified. Compared to previous work on metabolome profiling that typically reports less than 100 amine- and phenol-containing metabolites^17^,^20^, dansylation labeling LC-MS achieved a much higher coverage for this targeted sub-metabolome.

A comparison between non-injured controls and the three injury groups revealed a number of metabolites that show significant alterations induced by SCI. In particular, it was found that uridine was down-regulated considerably in both CSF and serum. Uridine is an important metabolic precursor of phosphotidylcholine, a major class of the cell membrane phospholipids that plays a key role in cell growth and repair^21^. Another metabolic function of uridine is to preserve ATP through anaerobic glycolysis^22^. Therefore, uridine can act as an alternative energy source when glucose supply is not sufficient, or under hypoxic and/or ischemic conditions. Although a direct link between uridine and SCI has not been reported, our findings suggest that the decrease in uridine level may be associated with several pathophysiological consequences of SCI, including membrane damage, phospholipase activation, hypoxia and ischemia^23^.

Subsequent pathway analysis of these differentially expressed metabolites indicated a perturbation in the arginine and proline metabolism. Among the 11 metabolites detected in this study, 6 (citrulline, arginine, proline, N-acetylputrescine, Gamma-aminobutyric acid and homocarnosine) and 3 (sarcosine, N-acetylputrescine and 4-hydroxyproline) of them were found to be significantly altered in at least one injury group in CSF and serum. Analysis of the CSF arginine and proline pathway revealed a significant decrease in arginine and increases in a few downstream metabolites towards the synthesis of homocarnosine. Arginine is the precursor of the biologically active molecule, nitric oxide (NO), through a reaction catalyzed by the nitric oxide synthase (NOS) family. NO is known to play a key role in the pathogenesis of acute CNS injury^24^. A competing reaction to NO production is catalyzed by arginase enzymes to generate ornithine and urea. It has been shown that following spinal cord injury, there is a significant increase in the expression of NOS2 mRNA^25^ and in the inducible NOS (iNOS) activity^24^. In addition, an increased arginase activity has also been reported to serve as a regulation of NO production, since high NO levels are known to be neurotoxic^26^. Activation of both types of enzymes would result in a lower arginine level in patients with SCI. In contrast, a significant increase of the final metabolic product, homocarnosine, has been observed in all three injury groups. Homocarnosine is a dipeptide composed of L-histidine and GABA, and is well-known for its antioxidant and neuroprotective activity^27^,^28^,^29^. Reactive oxygen species and oxidative stress are often considered as hallmarks of SCI^30^, and the enhanced production of homocarnosine is likely intended to mitigate such oxidative stress conditions.

The metabolic changes associated with serum arginine and proline metabolism are quite different from that of CSF. For example, in contrast to CSF, we found that arginine levels in the serum did not change significantly. In addition, the brain-specific metabolite homocarnosine could not be detected in serum. On the other hand, metabolites that exhibited significant alterations in serum, such as 4-hydroxyproline and sarcosine, did not show any difference between non-injured and injured CSF samples. This observation highlights the importance of performing parallel metabolomic profiling of serum and CSF at the discovery stage. While there were many common metabolites in serum and CSF, the metabolic responses to SCI were found to be very different. The immediate proximity of the CSF to the injured spinal cord makes it the most direct reflection of injury responses within the cord; accessing CSF, however, is obviously far more challenging than obtaining blood samples for biomarker studies. A parallel analyses such as ours enables one to compare the responses in both biofluids and determine how well processes within the CSF are manifested in the serum.

From the clinical perspective, it would be of great value to establish specific metabolites that can accurately classify injury severity, especially in situations where the functional measures are difficult to assess and are poor predictors of outcome (e.g., acute clinical trials). In this study, we identified six CSF metabolites and one serum metabolite that show promise as markers for evaluation of SCI injury severity. This targeted metabolic profiling result promotes the use of CSF for severity analysis because of the greater number of differentially expressed metabolites within CSF. For these six CSF metabolites, the metabolite levels were the highest in the AIS A group, and followed the trend A > B > C. This observation is consistent with the notion that the AIS A group represents the greatest injury severity (motor and sensory complete paralysis) and the severity level lowers from B (motor complete, sensory incomplete paralysis) to C (incomplete motor and sensory paralysis). Therefore, we would expect the greatest metabolic disturbance in AIS A. By using a combination of these metabolites, we have shown that good classification performance can be achieved in pair-wise comparisons (i.e., AUC > 0.8). In addition, some of the metabolic changes can be rationalized by considering their biological functions.

Firstly, citrulline is produced from arginine, and is the by-product of the reaction catalyzed by NOS that releases NO. As described earlier, activation of the NOS family following SCI would promote generation of NO as well as the by-product citrulline. On the other hand, the NO and citrulline levels are also regulated by activation of arginase, an enzyme that catalyzes a competing reaction and consumes arginine. While a decrease in arginine has been consistently detected in all three injury groups, the production of citrulline was found to be severity-dependent. For patients associated with “complete” paralysis (AIS A), the elevation in citrulline level is more pronounced compared to AIS B and C. On the other hand, patients in the AIS C category did not show significant change in citrulline level compared to the non-injured controls. This observation suggests that regulation of these two enzyme families (NOS and arginase) is related to the injury severity.

We have also identified glycerol and lactate to be differentially expressed among three injury groups. Glycerol is the end-product of phospholipid degradation. Activation of phospholipases A and C is known to occur after traumatic brain or spinal cord injury, which is thought to be associated with an increase in intracellular free Ca^2+^ 31. As an evidence of increased phospholipase activity, an injury severity-dependent decrease in the total lipid phosphorus level in rat spinal cord has been reported at 15 min, 4 hr and 24 hr after injury^31^. Consequently, the phospholipid breakdown product glycerol is expected to accumulate. Indeed, it has been shown that there was a significant increase in interstitial glycerol level immediately following trauma on the rat brain, and it was concluded that interstitial glycerol could serve as a marker for phospholipid degradation in acute brain injury^32^. Our observation of the increased glycerol level is also consistent with this mechanism. Lactate is a marker for hypoxia, as it provides an alternative pathway for energy production in the absence of oxygen. Increased CSF lactate levels have been reported on Days 1 through 9 following experimental spinal cord injury on cat models, indicating continuing hypoxia in the spinal cord tissue^33^. Similarly, a rise in interstitial lactate has also been observed in the brain injury of rats^32^,^34^. In this work, we have shown that an elevated level of CSF lactate is also present in human patients, and the response is related to injury severity. This is also consistent with our previous work in a pig model of SCI in which spinal cord microdialysis demonstrated elevated lactate levels as well^35^.

Another metabolic change that is worth noting is the elevation of two acetylated polyamines, N-acetylputrescine and N1, N12-diacetylspermine, following acute SCI. In particular, N1, N12-diacetylspermine is not detected in the non-injured samples, but is highly elevated in the AIS A group. Acetylation of polyamines is catalyzed by spermidine/spermine N1–acetyltransferase (SSAT) as part of the polyamine catabolism process. Although the relationship between polyamine acetylation and spinal cord injury has not been extensively reported, a recent study demonstrated an enhanced polyamine catabolism, including acetylation, after traumatic brain injury^36^. In the above-mentioned study, it was shown that N1-acetylspermidine was detectable 24 and 72 hr post injury, indicating an increased SSAT activity and disrupted polyamine homeostasis. Our finding of the elevated acetylated polyamines indicates that the same mechanism may also occur in the acute SCI scenario.

In summary, six identified CSF metabolites have emerged as potential biomarkers for assessment of injury severity, and good classification performance has been achieved by using a combination of these metabolites in pair-wise comparisons of AIS A, B and C patients. In addition, we have shown that using significantly changed metabolites that have not been positively identified at this stage can further improve the prediction accuracy. For future work, more samples will be used to validate these potential biomarkers as well as examine the possibility of finding biomarkers that predict neurologic recovery or that could be used as surrogate outcome measures. In this regard, the use of UMS allows us to readily expand the current dataset by analyzing new samples. Because the same or comparable ^13^C-UMS will be used to analyze new batches of samples, the peak ratio values obtained from these individual samples will be fully compatible to those shown in this study^12^.

## Materials and Methods

### Clinical Sample Collection

Patients with acute traumatic SCI were enrolled in a prospective observational trial to obtain CSF, entitled CAMPER (the Canadian Multicenter CSF Pressure and Biomarker Study). Participating sites included Vancouver (British Columbia), Montreal (Quebec), Halifax (Nova Scotia) and London (Ontario). The CAMPER trial is registered with ClinicalTrials.gov (Registration number: NCT01279811; Registration date: January 18, 2011) and is conducted in accordance to the Declaration of Helsinki ethical principles for medical research involving human subjects. The CAMPER clinical trial protocol and experimental procedures (see CAMPER Protocol in additional review material) received ethics approval from each participating center’s Institutional Review Board (IRB): Vancouver site from University of British Columbia Clinical Research Ethics Board, London site from Western University Health Sciences Research Ethics Board, Montreal site from Hôpital du Sacré-Coeur de Montréal Ethics Committee of Research and Halifax site from Capital Health Research Ethics Board. Additional sites including San Francisco and Stanford have been added to enroll more patients for future research; no samples from these new sites were used in this current study. Ethics approval was received from the University of California San Francisco, Human Research Protection Program Committee on Human Research and Stanford University Institutional Review Board. Each SCI patient provided his/her informed consent for the study. Intrathecal catheters were inserted pre-operatively into 30 acute SCI patients with baseline ASIA Impairment Scale (AIS) grades of A (n = 10), B (n = 12) and C (n = 8). These catheters were left in place for 5 days, during which CSF/serum samples were drawn approximately every 8 hrs. The CSF and serum samples were centrifuged at the bedside and the supernatant was immediately frozen on dry ice. The collected samples were shipped on dry ice and stored at −80 °C until further use. For each patient, three CSF and serum samples drawn at different time points were used for this study. These samples were grouped into three approximate daily stages: 24 hours (t_1_, 16–32 hours), 48 hours (t_2_, 40–56 hours) and 72 hours post-injury (t_3_, 64–80 hours).

### Metabolite Extraction and Labeling

Metabolites were extracted from serum and CSF samples via methanol protein precipitation. Three volumes of ice-cold methanol were added into 25 μL of serum/CSF, vortexed and incubated on ice for 15 min. This was followed by centrifugation at 20 817 g for 15 min. The supernatants (75 μL for serum and 90 μL for CSF) were dried using a SpeedVac and resuspended in 25 μL of water. For the labeling step using the dansyl chloride agents available at MCID.chem.ualberta.ca, the extracted solution was mixed with 12.5 μL 250 mM sodium carbonate/sodium bicarbonate buffer and 12.5 μL ACN. The solutions were vortexed, spun down and mixed with 25 μL freshly prepared ^12^C-dansyl chloride solution (18 mg/mL) (for light labeling) or ^13^C-dansyl chloride solution (18 mg/mL) (for heavy labeling). The reaction was allowed to proceed for 1 hr at 40 °C. After 1 hr, NaOH (250 mM, 5 μL) was added to the reaction mixture to quench the excess dansyl chloride. The solution was then incubated at 40 °C for another 10 min. Finally, formic acid in 50/50 ACN/H_2_O (425 mM, 25 μL) was added to consume excess NaOH and to make the solution acidic.

### Preparation of Labeled Samples for LC-MS Analysis

In the analysis of analytical variability, a 90 μL aliquot was taken from one CSF sample of each patient group (AIS A, B, C and healthy control). Three of the 25 μL portions from each sample were labeled with ^12^C-dansyl chloride as three experimental replicates, while the remaining liquids were mixed together to generate a pooled sample which was subsequently labeled by ^13^C-dansyl chloride. The same procedure was performed for serum samples. In all other studies, aliquots of all individual CSF/serum samples were mixed together to generate a pooled CSF/serum sample. For comparison between the serum and CSF metabolome, equal aliquots of pooled CSF and serum were combined and labeled with ^13^C-dansyl chloride, while the pooled CSF and pooled serum samples were labeled with ^12^C-dansyl chloride. For metabolomic profiling of the entire sample set, all individual samples were labeled with ^12^C-dansyl chloride, and the pooled CSF and pooled serum samples were labeled with ^13^C-dansyl chloride. Prior to LC-MS analysis, the ^12^C-labeled samples were combined with an equal amount of the corresponding ^13^C-labeled reference. Quality control (QC) samples were prepared by 1:1 volume mix of a ^12^C-labeled and a ^13^C-labeled pooled sample.

### LC-MS Analysis and Data Processing

The labeled metabolites were analyzed using a Bruker Maxis Impact QTOF mass spectrometer (Bruker, Billerica, MA) linked to an Agilent 1100 series binary HPLC system (Agilent, Palo Alto, CA). The samples were injected onto an Agilent reversed phase Eclipse Plus C18 column (2.1 mm × 10 cm, 1.8 μm particle size, 95 Å pore size) for separation. Solvent A was 0.1% (v/v) formic acid in 5% (v/v) acetonitrile, and solvent B was 0.1% (v/v) formic acid in acetonitrile. The chromatographic conditions were: t = 0 min, 20% B; t = 3.5 min, 35% B; t = 18 min, 65% B; t = 24 min, 99% B; t = 31 min, 99% B, t = 32 min, 20% B. The flow rate was 180 μL/min. All MS spectra were obtained in the positive ion mode with a scan range of 150 to 1000 m/z. The capillary voltage was 4500 V and nebulizer pressure was 1.8 bar. The dry gas flow was set to 8 l/min and the dry gas temperature was set to 230 °C. Quality control samples were analyzed between every 20 sample runs to monitor instrument performance. For each LC-MS run, masses were calibrated to the spectrum that contained the dansyl-amine peaks at m/z 242.57160 (two tags two charges), m/z 484.13592 (two tags one charge) and m/z 971.27799 (dimer) using the Data Analysis software and the calibration result was applied to all the other spectra in the same LC-MS run. The resulting MS data were processed using a peak-pair picking software, IsoMS^37^. The level 1 peak pairs were aligned from multiple runs by retention time within 20 s and accurate mass within 5 ppm. Metabolite identification was based on accurate mass and retention time search against the dansyl standard library with mass difference of less than 5 ppm and retention time shift of less than 30 s. Dansyl library search does not require MS/MS for positive identification, as accurate mass and retention time matches are already sufficient^14^. It should be noted that in the list of identified metabolites, imidazole acetic acid has an amine group that can be labeled by dansylation as expected. For some conjugated alcohols, the hydroxyl group behaves like a phenol group and thus can be labeled by dansylation. Lactic acid, glycerol and several common sugar molecules such as glucose belong to this type of alcohols and can be labeled.

### Statistical Analysis

Only those peak-pair features shared by more than 50% of the samples were retained for statistical analysis. Multivariate statistical analysis including principle component analysis (PCA) and partial least squares discriminant analysis (PLS-DA) was carried out using SIMCA-P+ 12.0 (Umetrics, Umeå, Sweden). PLS-DA validation was performed using 20-permutation test built in the SIMCA-P+ 12.0 program. The threshold for selection of significant features in PLS-DA was VIP > 1.5. Analysis of variance (ANOVA) and Tukey’s test were performed by Metaboanalyst (www.metaboanalyst.ca)^38^ with *p* < 0.05. For comparisons between two groups, the fold change and *p*-value by Student’s *t* test were calculated using Microsoft Excel. The multiple-testing-corrected *p*-value (*q*-value) was calculated using R and BioConductor (www.bioconductor.org)^39^. The data were mean-centered and auto-scaled (unit variance) prior to analysis. ROC analysis was performed using SPSS (IBM Corp., Armonk, NY, USA). Predictions based on logistic regression model were performed by Waikato Environment for Knowledge Analysis (Weka, University of Waikato)^40^. Metabolites with *p* < 0.05 from the analysis of variance were ranked using the greedy stepwise approach. The prediction model was established in two steps, and leave-one-out cross validation (LOOCV) was utilized to establish the prediction accuracy. Firstly, logistic regression was applied for predicting AIS A vs. non-AIS A on all samples (for each sample, a logistic regression model was built based on all the other samples, and the modeling result was used to evaluate this left out sample). Then, the predicted non-AIS A underwent a second logistic regression LOOCV for predicting AIS C vs. non-AIS C. In this step, all non-AIS Cs would be assigned as AIS B. In each step, the optimal number of top ranked metabolites for building prediction models was determined based on the highest prediction accuracy, with a restriction of 6 metabolites at maximum. The final logistic regression equation has been calculated using all of the samples involved in that step. A 3 × 3 confusion matrix table was constructed, and the overall prediction accuracy was calculated as the percentage of samples that have been correctly assigned as A, B or C (i.e., along the diagonal of the confusion matrix table).

## Additional Information

**How to cite this article**: Wu, Y. *et al*. Parallel Metabolomic Profiling of Cerebrospinal Fluid and Serum for Identifying Biomarkers of Injury Severity after Acute Human Spinal Cord Injury. *Sci. Rep.* **6**, 38718; doi: 10.1038/srep38718 (2016).

**Publisher's note:** Springer Nature remains neutral with regard to jurisdictional claims in published maps and institutional affiliations.

## Supplementary Material

Supplementary Information

## Figures and Tables

**Table 1:** List of significantly altered metabolites between injured and non-injured CSF and serum samples.

CSF metabolites	A VS. N			B VS. N			C VS. N		
	*p*-value	FC	*q*-value	*p*-value	FC	*q*-value	*p*-value	FC	*q*-value
Uridine	**2**.**74E-08**	**0**.**29**	**6**.**82E-07**	**1**.**39E-09**	**0**.**25**	**3**.**12E-08**	**2**.**83E-04**	**0**.**37**	**0**.**00523**
Imidazoleacetic acid	**2**.**09E-04**	**0**.**45**	**0**.**00149**	**0**.**00322**	**0**.**56**	**0**.**0206**	**0**.**00527**	**0**.**52**	**0**.**0406**
Methionine Sulfoxide	**1**.**25E-05**	**0**.**46**	**1**.**56E-04**	**8**.**72E-05**	**0**.**50**	**0**.**00131**	**1**.**22E-04**	**0**.**43**	**0**.**00300**
Arginine	**2**.**97E-06**	**0**.**50**	**4**.**93E-05**	**0**.**00164**	**0**.**62**	**0**.**0123**	**0**.**00177**	**0**.**59**	**0**.**0206**
Cystathionine	**0**.**0180**	**3**.**78**	**0**.**0386**	**0**.**00373**	**3**.**07**	**0**.**0209**	**0**.**0139**	**2**.**63**	**0**.**0770**
Homocarnosine	**0**.**00582**	**3**.**79**	**0**.**0159**	**0**.**00493**	**3**.**22**	**0**.**0212**	**0**.**0186**	**3**.**43**	**0**.**0899**
Alpha-aminobutyric acid	**0**.**0157**	**1**.**68**	**0**.**0355**	**0**.**00153**	**1**.**90**	**0**.**0123**	0.0312	1.44	0.121
N-Acetylputrescine	**3**.**69E-04**	**1**.**92**	**0**.**00153**	**0**.**00568**	**1**.**58**	**0**.**0212**	0.0982	1.28	0.220
N-methyl-D-aspartic acid	**0**.**00328**	**2**.**34**	**0**.**00961**	**0**.**0301**	**1**.**54**	**0**.**0644**	0.546	0.91	0.615
Lysine	**3**.**08E-04**	**0**.**53**	**0**.**00153**	0.00657	0.69	0.0227	**3**.**71E-05**	**0**.**58**	**0**.**00274**
2-Aminobenzoic acid	**0**.**00104**	**1**.**60**	**0**.**00345**	0.0755	1.51	0.130	**0**.**00195**	**1**.**53**	**0**.**0206**
Proline	**6**.**35E-04**	**3**.**54**	**0**.**00243**	0.140	6.66	0.159	**0**.**0146**	**3**.**09**	**0**.**0770**
2-Phenylglycine	0.297	0.81	0.242	**0**.**0164**	**0**.**65**	**0**.**0433**	**0**.**0200**	**0**.**48**	**0**.**0899**
Ethanolamine	**3**.**86E-05**	**0**.**59**	**3**.**20E-04**	0.0271	0.70	0.0608	0.0124	0.69	0.0763
Citrulline	**0**.**0286**	**1**.**58**	**0**.**0526**	0.356	1.18	0.250	0.336	0.83	0.469
Cadaverine	**0**.**0143**	**1**.**64**	**0**.**0339**	0.154	2.30	0.162	0.773	0.96	0.715
Glycylproline	**0**.**0277**	**1**.**73**	**0**.**0526**	0.145	1.60	0.159	0.243	1.23	0.408
Lactic acid	**1**.**83E-05**	**2**.**12**	**1**.**82E-04**	0.0221	1.49	0.0522	0.0307	1.29	0.121
Glycerol	**2**.**87E-04**	**2**.**48**	**0**.**00153**	0.0550	1.52	0.112	0.238	1.24	0.408
Oxidized glutathione	**0**.**0194**	**3**.**58**	**0**.**0386**	0.102	1.99	0.150	0.231	2.61	0.408
Gamma-Aminobutyric acid	0.188	1.67	0.184	**0**.**0155**	**2**.**11**	**0**.**0433**	0.126	1.58	0.266
Threonine	6.07E-03	0.68	0.0159	0.0957	0.81	0.148	**0**.**00610**	**0**.**65**	**0**.**0410**
Isoleucine	6.85E-02	0.60	0.0948	0.0923	0.63	0.148	**0**.**0484**	**0**.**62**	**0**.**162**
5-Hydroxyindoleacetic acid	1.29E-01	1.36	0.153	0.0693	1.52	0.124	**0**.**0458**	**1**.**96**	**0**.**162**
**Serum metabolites**	**A VS. N**			**B VS. N**			**C VS. N**		
	***p*-value**	**FC**	***q*-value**	***p*-value**	**FC**	***q*-value**	***p*-value**	**FC**	***q*-value**
Uridine	**1**.**90E-05**	**0**.**43**	**5**.**01E-04**	**1**.**27E-06**	**0**.**41**	**3**.**56E-05**	**1**.**34E-05**	**0**.**35**	**4**.**86E-04**
4-Hydroxyproline	**0**.**00384**	**0**.**62**	**0**.**0221**	**0**.**0108**	**0**.**65**	**0**.**0757**	**0**.**0210**	**0**.**63**	**0**.**0951**
N1, N12-Diacetylspermine	**0**.**00163**	**2**.**06**	**0**.**0145**	**0**.**00846**	**1**.**96**	**0**.**0757**	**0**.**0364**	**1**.**63**	**0**.**134**
Glycylproline	**1**.**88E-04**	**3**.**20**	**0**.**00372**	**0**.**0421**	**2**.**51**	**0**.**169**	**1**.**59E-04**	**3**.**09**	**0**.**00230**
Sarcosine	**0**.**00670**	**0**.**54**	**0**.**0332**	0.0644	0.68	0.208	**0**.**0135**	**0**.**58**	**0**.**0753**
Phenylalanine	**1**.**21E-05**	**1**.**60**	**4**.**79E-04**	6.38E-06	1.43	1.34E-04	**5**.**92E-05**	**1**.**56**	**0**.**00107**
2-Aminooctanoic acid	0.0489	0.67	0.125	**0**.**00138**	**0**.**55**	**0**.**0194**	**0**.**0256**	**0**.**56**	**0**.**109**
5-Hydroxylysine	0.199	1.14	0.323	**0**.**0237**	**1**.**51**	**0**.**127**	**0**.**00361**	**1**.**72**	**0**.**0262**
Hydroxyphenyllactic acid	**0**.**00341**	**1**.**56**	**0**.**0221**	0.0159	1.48	0.0956	0.137	1.31	0.333
Guaiacol	**0**.**0120**	**1**.**59**	**0**.**0545**	0.170	1.22	0.353	0.806	1.06	0.664
N-Acetylputrescine	**0**.**0271**	**2**.**46**	**0**.**0825**	0.0887	2.18	0.228	0.0712	1.80	0.206
Vanillylmandelic acid	0.00506	1.50	0.125	**0**.**0106**	**1**.**50**	**0**.**0757**	0.624	1.11	0.595
Alpha-aminobutyric acid	0.0192	1.44	0.0675	**7**.**93E-04**	**1**.**80**	**0**.**0133**	0.00362	1.49	0.0262

Fold change (FC) and *p*-value that met the selection criteria (fold change >1.5, *p* < 0.05) are shown in bold.
