# Supplementary material for: Parallel Metabolomic Profiling of Cerebrospinal Fluid and Serum for Identifying Biomarkers of Injury Severity after Acute Human Spinal Cord Injury
Source: Sci Rep. 2016 Dec 14;6:38718. doi: 10.1038/srep38718 (PMC5155264; doi:10.1038/srep38718)
Supplement: Supplementary Information [file srep38718-s1.pdf]

# Supplemental Information

# Parallel Metabolomic Profiling of Cerebrospinal Fluid and Serum for Identifying Biomarkers of Injury Severity after Acute Human Spinal Cord Injury

Yiman Wu[1], Femke Streijger[2], Yining Wang[3], Guohui Lin[3], Sean Christie[4], Jean-Marc Mac-Thiong[5], Stefan Parent[6], Christopher S. Bailey[7], Scott Paquette[8], Michael C. Boyd[8], Tamir Ailon[8], John Street[9], Charles G. Fisher[9], Marcel F. Dvorak[9], Brian K. Kwon[2]*, Liang Li[1]*

1. Department of Chemistry, University of Alberta, Edmonton, AB T6G2G2, Canada
2. International Collaboration on Repair Discoveries (ICORD), University of British Columbia, Blusson Spinal Cord Centre, 818 West 10th Avenue, Vancouver, BC, V5Z 1M9, Canada
3. Department of Computing Science, University of Alberta, Edmonton, AB, T6T 2E8, Canada
4. Division of Neurosurgery, Dalhousie University, Halifax Infirmary, 1796 Summer Street, Halifax, NS, B3H 3A7, Canada
5. Hôpital du Sacré-Coeur de Montréal, 5400 Boul Gouin O, Montréal, QC, H4J 1C5, Canada
6. Chu Sainte-Justine, Dept. of Surgery, Université de Montréal, PO Box 6128, Station Centre-ville, Montreal, QC, H3C 3J7, Canada
7. Division of Orthopaedic Surgery, Schulich Medicine & Dentistry, Victoria Hospital 800 Commissioners Road East, Room E4 120, London, ON, N6C 5W9, Canada
8. Division of Neurosurgery, University of British Columbia, Vancouver Spine Surgery Institute, 818 West 10th Avenue, Vancouver, BC, V5Z 1M9, Canada
9. Department of Orthopaedics, University of British Columbia, Vancouver Spine Surgery Institute, 818 West 10th Avenue, Vancouver, BC, V5Z 1M9, Canada

# Supplemental Note S1. CIL LC-MS Metabolomic Profiling and Performance Evaluation

## 1. Overview of metabolomic profiling workflow

Figure 1 in the main text illustrates the overall differential isotope labeling metabolomic profiling workflow. In this workflow, CSF and serum samples were analyzed in parallel. CSF and serum metabolites were extracted via protein precipitation with three volumes of MeOH. After extraction, the total solution volume is 100 μL. However, because serum contains a large amount of protein precipitates, in practice, we recommend that only 75 μL of the serum supernatant to be taken in order to avoid the precipitate. On the other hand, the protein content in CSF is small, and therefore 90 μL of the CSF supernatant can be taken without having the precipitate in the solution. Prior to LC-MS analysis, each $^{12}$C-labeled individual CSF or serum sample was combined with an equal amount of the corresponding $^{13}$C-labeled pooled reference sample. The LC-MS data was processed by IsoMS[1] to extract peak ratio information for each individual peak pair found in the mass spectra, and the missing values were retrieved using the zero-fill program[2]. Finally the processed data was subjected to statistical analysis for discovery of differentiating metabolites. Since each sample was only analyzed once (i.e., no experimental replicates), it is important to ensure a good peak detectability and repeatability of this analytical platform. Therefore, these parameters were first examined prior to the metabolomic profiling analysis.

## 2. Evaluation of the Analytical Platform

To evaluate the analytical variability, the coefficient of variation (CV) was determined from three experimental replicates that is a combination of variations introduced during protein

precipitation, labeling, solution mixing, LC-MS detection and data processing. The CV values were calculated by using peak ratios of all peak pairs commonly detected in three replicate runs. Table 1 lists the median and average CV values for each sample. For all eight samples, the median CVs were less than 15% and the mean CVs were of 17% or less. In addition to determination of the CV values, we also examined the percentage of commonly detected peak pairs from experimental triplicates. The non-common peak pairs, or missing values, in replicate runs are usually caused by the presence of borderline metabolites, false positive peak pair identification, or other situations in which the peak picking criteria are not met (e.g., large retention time or mass shift). Therefore, the percentage of common peak pairs reflects both the reproducibility of measurements and the robustness of the data analysis procedure. Figure 1 shows the number distributions of the peak pairs found in three experimental replicates for all eight samples. The percentage overlap of detected metabolites between three experimental replicates was over 92% for all samples examined, suggesting a good reproducibility of this analytical platform. The analytical variability is very similar in CSF and serum based on this differential isotope labeling approach.

Table 1. Measured analytical variations in CSF and serum samples

| Biofluid | Sample group | Mean CV (%) | Median CV (%) |
|---|---|---|---|
| **CSF** | AIS A | 17 | 12 |
| | AIS B | 13 | 11 |
| | AIS C | 17 | 14 |
| | Control | 16 | 14 |
| **Serum** | AIS A | 16 | 12 |
| | AIS B | 17 | 14 |
| | AIS C | 16 | 12 |
| | Control | 17 | 14 |

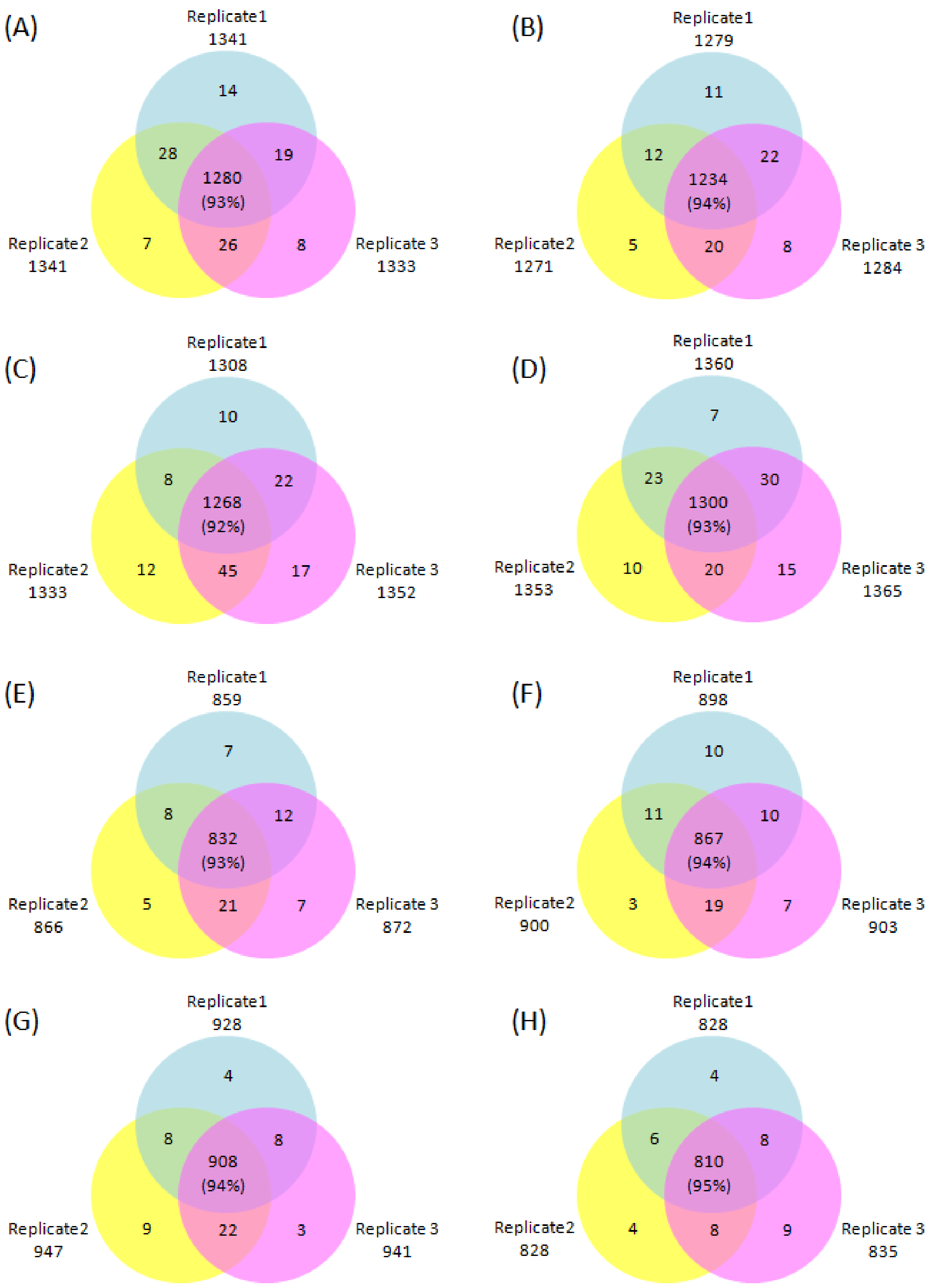


Figure 1. Distributions of the number of peak pairs detected in three experimental replicates for (A) serum AIS A; (B) serum AIS B; (C) serum AIS C; (D) serum control; (E) CSF AIS A; (F) CSF AIS B; (G) CSF AIS C; (H) CSF control.

In LC-MS analysis, the sample injection amount plays an important role on the number of metabolites detected. For small injection amounts, metabolites with concentrations close to the instrument detection threshold (known as borderline metabolites)[3] may not be detected. On the other hand, with large injection amounts, signals from low abundance metabolites co-eluting with a high intensity metabolite may be suppressed. Moreover, column saturation and sample carryover problems may occur. In this work, we examined the relationship between the number of peak pairs detected and the injection volume using the pooled CSF and serum samples (Figure 2). The injection amount can be calculated by multiplying the injection volume with the nominal total metabolite concentrations of CSF and serum determined from the calibration curve established with a mixture of 17 amino acid standards[4,5]. For CSF, the number of peak pairs increases by 20% when the injection volume increases from 6 μL to 12 μL, and then levels off. This significant increase in the number of peak pairs is likely attributed to the low metabolite concentration levels in CSF samples, since many of the borderline metabolites cannot be detected when the injection amount is not sufficiently high. In contrast, by increasing the injection volume from 6 μL to 10 μL, the number of peak pairs increases only by 5%, and further increase in the injection volume leads to a decrease in the peak pair number. Compared to CSF, the metabolite levels in serum are considerably higher, and consequently the percentage of borderline metabolites is smaller. On the other hand, at higher injection amounts, ion suppression from high abundance ions becomes noticeable in serum samples[6]. The effect of ion suppression at higher injection amounts is illustrated in Figure 3. In this example, the signals of the low abundance peak pair 318.560 and 320.567 increases when the injection volume increases from 6 μL to 8 μL, and decreases as the injection volume increases further. At an injection volume of 14

μL, this peak pair falls below the detection threshold and becomes non-detectable. Considering both effects (borderline metabolites and ion suppression), the optimal injection amount was chosen as the point at which the number of peak pairs levels off or starts to decrease. Based on Figure 3, the optimal injection volume was determined to be 12 μL for CSF and 10 μL for serum (the corresponding injection amount was 3.3 nmole and 5.7 nmole), which gave 1213 and 2316 peak pairs for CSF and serum, respectively. It is not surprising to see a smaller number of metabolites in CSF compared to serum. As the primary carrier of small molecules in the body, the metabolite composition in serum is much more complex than CSF, and it also contains a greater number of exogenous compounds.

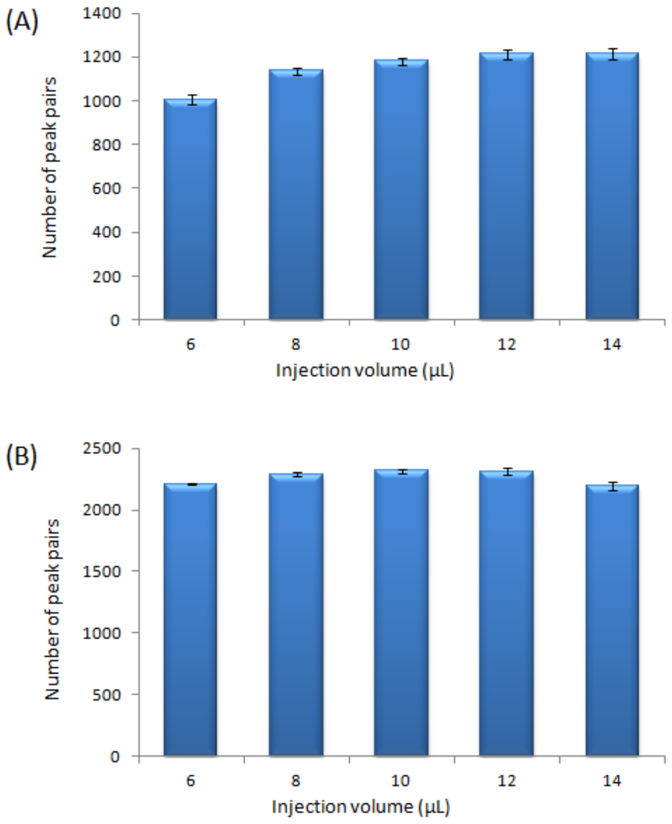


Figure 2. Plot of the number of peak pairs detected against the injection volume for (A) CSF and (B) serum.

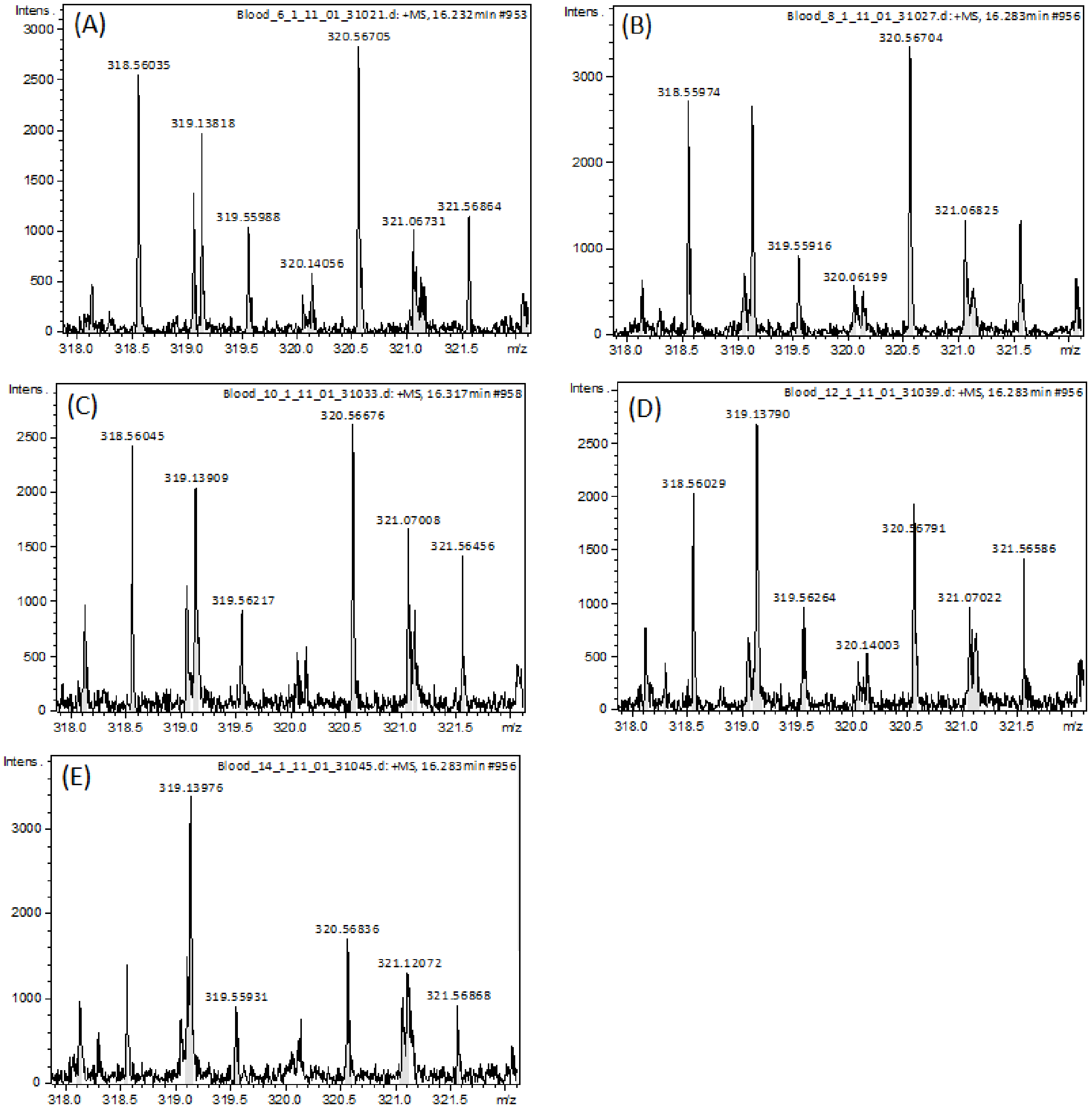


Figure 3. An example showing ion suppression effect with larger injection amounts for the peak pair 318.560 and 320.567. The injection amounts are (A) 6 µL; (B) 8 µL; (C) 10 µL; (D) 12 µL and (E) 14 µL.

## 3. Comparison of the CSF and Serum Metabolome

Figure 4 shows the overlaid base-peak ion chromatograms (BPCs) for CSF and serum. Most of the high abundance peaks correspond to labeled amino acids (indicated by stars). It is noted that while the majority of the peaks were common to CSF and serum, the metabolite intensity in serum is much higher than that in CSF. For example, among the fifteen identified amino acids in Figure 4, only glutamine gives higher signal in CSF compared to serum, while all the other fourteen amino acids have much higher abundance in serum. In addition, since serum is a lipid rich biofluid, we also observed several phospholipid peaks in the BPC of serum (labeled with dots), which were barely detectable in CSF. These unlabeled compounds appear as singlet peaks in the mass spectra and therefore will not be considered in the current metabolomic profiling workflow.

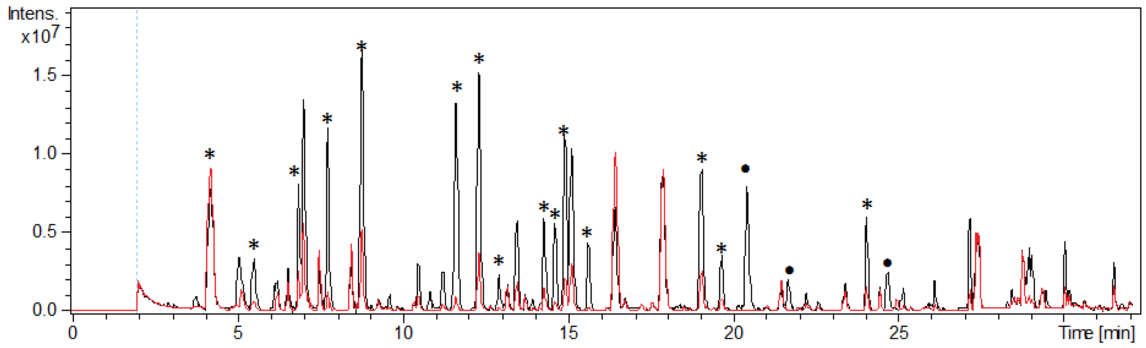


Figure 4. Base-peak ion chromatograms of CSF (red) and serum (black). Peaks labeled with a star correspond to amino acids, and peaks labeled with a dot correspond to phospholipids. The first amino acid peak (eluted out at 4 min) is glutamine.

The BPC only shows metabolites with the greatest signal intensities. To compare the relative quantities of all metabolites common to CSF and serum, a “pooled pool” reference sample was generated by combining the pooled CSF and serum samples and then labeled with $^{13}C$ dansyl chloride. The $^{12}C$-labeled CSF and serum pool samples were combined with the $^{13}C$-

labeled "pooled pool" reference in 1:1 volume ratio, so that the relative intensities of individual metabolites can be assessed based on the $^{12}C$ to $^{13}C$ peak ratios of each peak pair. Supplemental Figure 5 shows a scatter plot that compares the relative metabolite intensities in CSF and serum. For more than half of the peak pairs, the $^{12}C/^{13}C$ peak ratio is larger in serum than in CSF, indicating higher metabolite concentration in serum. In contrast, only less than 30% of the metabolites have higher concentrations in CSF. Moreover, there are a number of data points located along the y = x curve, which correspond to metabolites that have similar intensities in CSF and serum.

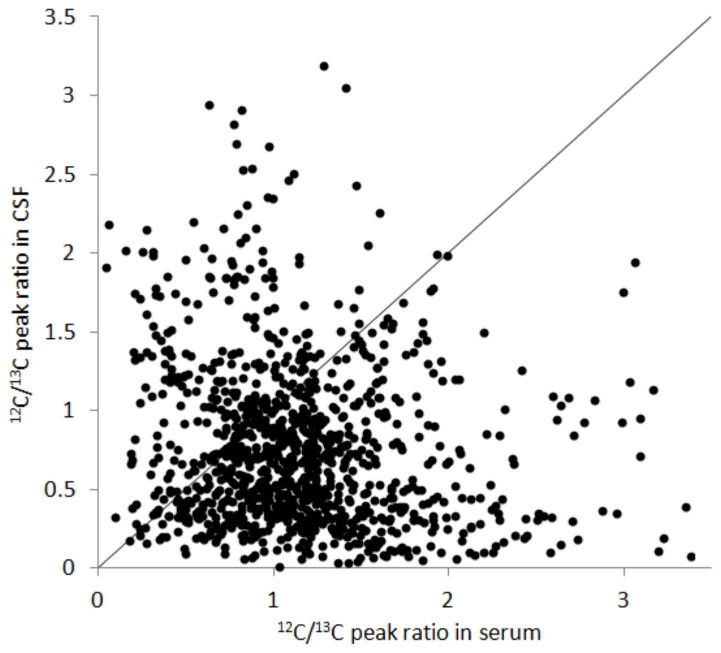


Figure 5. Comparison between the relative metabolite intensities in serum and CSF, expressed as $^{12}C/^{13}C$ peak ratios. Each dot represents a peak pair (or putative metabolite).

While CSF is often considered as an ideal biofluid for studying neurological disorders, the low metabolite concentration in CSF can pose a challenge to the biomarker discovery process, as the concentrations of some biologically meaningful metabolites may fall below the instrument

detection limit. For example, we found that serotonin, an important neurotransmitter, was not detected in CSF. On the other hand, the higher overall metabolite concentration in serum allows for more sensitive detection and identification of biomarkers. In addition, serum also has the advantage of easy accessibility, which makes it an attractive alternative for studying central nervous system disorders[7]. Nevertheless, some of the metabolites can only be detected in CSF (e.g., the brain-specific dipeptide homocarnosine). Also, since the matrix is more complex in serum, the detectability of low intensity metabolites can be compromised by ion suppression. For investigating metabolic changes associated with these compounds, analysis of CSF is the better approach. Therefore, the selection of the biofluid type plays an important role in the biomarker discovery process, and the choice is dependent on the objective of study, sample availability, instrument sensitivity and the metabolites of interest. Parallel metabolomic profiling of CSF and serum on a small pilot set may be carried out to assist selection of the biofluid.

**References**


1. Zhou, R., Tseng, C.L., Huan, T., & Li, L., IsoMS: automated processing of LC-MS data generated by a chemical isotope labeling metabolomics platform. *Anal Chem* 86 (10), 4675-4679 (2014).
2. Huan, T. & Li, L., Counting missing values in a metabolite-intensity data set for measuring the analytical performance of a metabolomics platform. *Anal Chem* 87 (2), 1306-1313 (2015).
3. Chen, Y. *et al.*, Combination of injection volume calibration by creatinine and MS signals' normalization to overcome urine variability in LC-MS-based metabolomics studies. *Anal Chem* 85 (16), 7659-7665 (2013).

4 Wu, Y. & Li, L., Determination of total concentration of chemically labeled metabolites as a means of metabolome sample normalization and sample loading optimization in mass spectrometry-based metabolomics. *Anal Chem* 84 (24), 10723-10731 (2012).

5 Xu, W. *et al.*, Development of high-performance chemical isotope labeling LC-MS for profiling the human fecal metabolome. *Anal Chem* 87 (2), 829-836 (2015).

6 Zhou, R.K. & Li, L., Effects of sample injection amount and time-of-flight mass spectrometric detection dynamic range on metabolome analysis by high-performance chemical isotope labeling LC-MS. *J. Proteomics* 118, 130-139 (2015).

7 Quinones, M.P. & Kaddurah-Daouk, R., Metabolomics tools for identifying biomarkers for neuropsychiatric diseases. *Neurobiol Dis* 35 (2), 165-176 (2009).

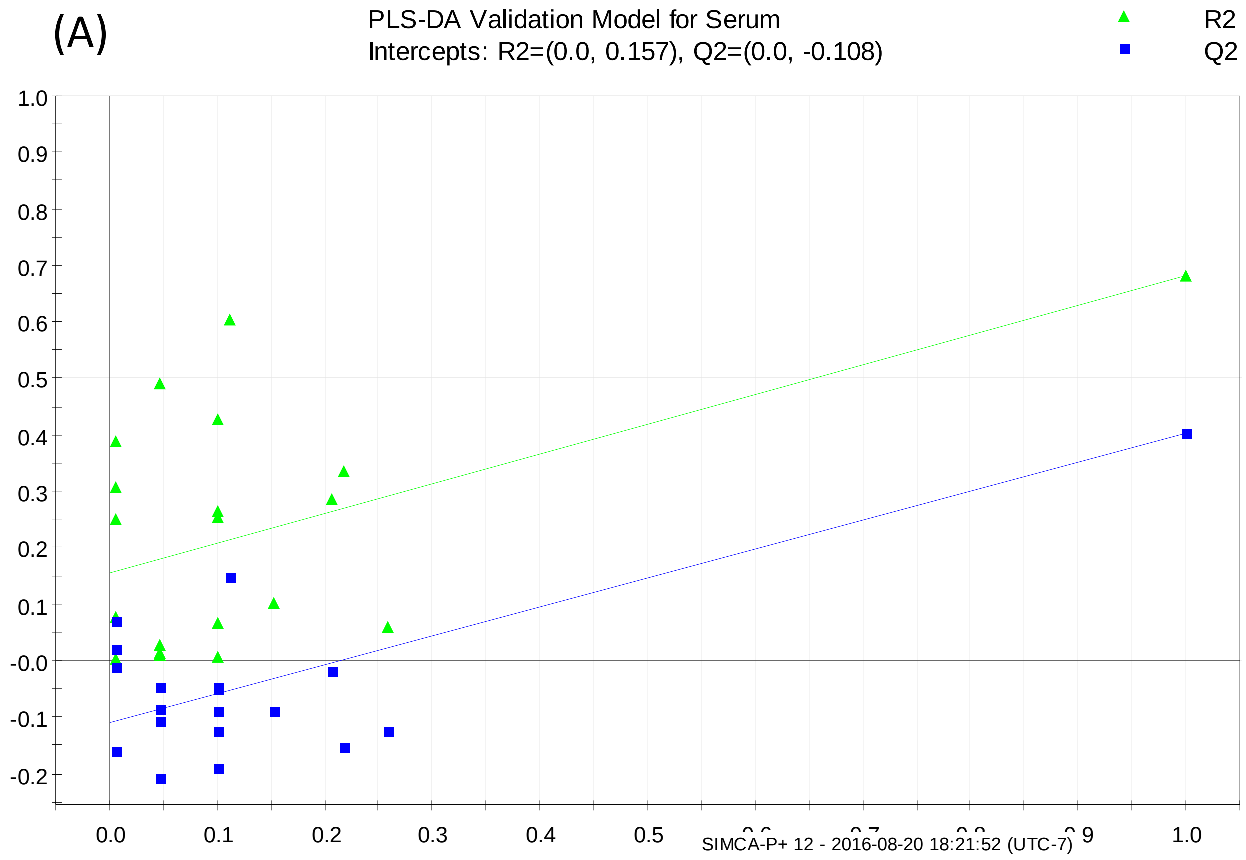


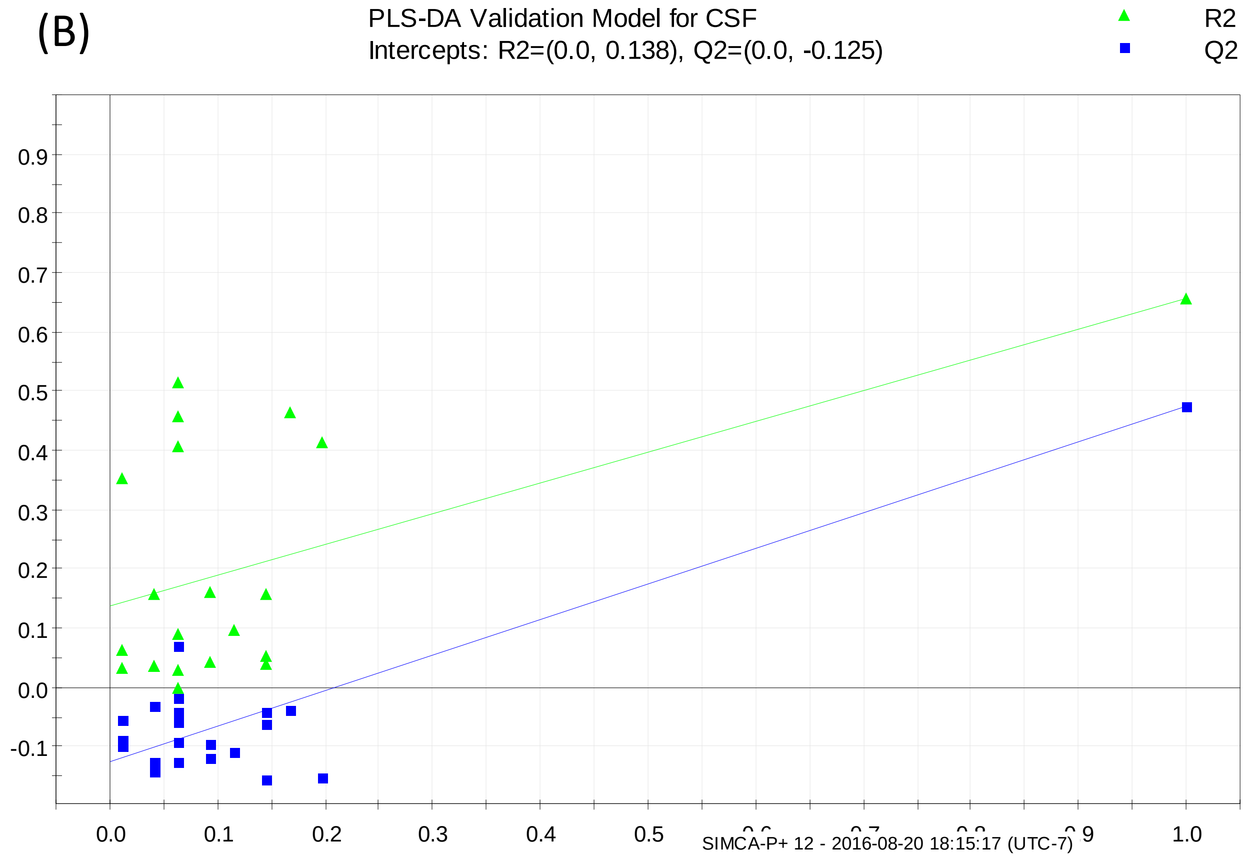


Supplemental Figure S1. Validation of PLS-DA models for (A) serum and (B) CSF data sets using twenty-permutation test.

Supplemental Tables T1-T8

Supplemental Table T1. List of metabolites positively identified based on retention time and mass matches to those of metabolite standards.

| HMDB.No. | Name | Accurate mass | mz_light | Retention time (min) | mz difference (ppm) | rt difference (s) | Note |
|---|---|---|---|---|---|---|---|
| HMDB01414 | 1,4-diaminobutane | 88.1000 | 278.1083 | 21.39 | 2.10 | 4.0 | |
| HMDB02362 | 2,4-Diaminobutyric acid | 118.0742 | 293.0954 | 15.85 | 0.45 | 5.5 | |
| HMDB01123 | 2-Aminobenzoic acid | 137.0477 | 371.1060 | 16.78 | 4.32 | 2.1 | |
| HMDB00991 | 2-aminooctanoic acid | 159.1259 | 393.1842 | 19.10 | 0.33 | 11.1 | |
| HMDB02210 | 2-Phenylglycine | 151.0633 | 385.1216 | 12.02 | 2.52 | 7.5 | |
| HMDB01336 | 3,4-Dihydroxybenzeneacetic acid | 168.0423 | 318.0794 | 23.86 | 2.34 | 3.0 | |
| HMDB03911 | 3-Aminoisobutanoic acid | 103.0633 | 337.1216 | 8.96 | 0.90 | 1.9 | |
| HMDB02390 | 3-Cresotinic acid | 152.0473 | 386.1057 | 17.06 | 2.37 | 8.1 | |
| HMDB00750 | 3-Hydroxymandelic acid | 168.0423 | 402.1006 | 13.37 | 0.17 | 14.8 | |
| HMDB00750_2 | 3-Hydroxymandelic acid - COOH | 168.0423 | 356.0951 | 21.80 | 1.34 | 7.0 | |
| HMDB00440 | 3-Hydroxyphenylacetic acid | 152.0473 | 386.1057 | 16.71 | 2.21 | 8.0 | |
| HMDB00022 | 3-Methoxytyramine or Phenylephrine | 167.0946 | 317.6056 | 25.50 | 0.39 | 1.7/7.7 | |
| HMDB00479 | 3-methyl-histidine or 1-Methylhistidine | 169.0851 | 403.1434 | 2.41 | 0.86 | 2.5/7.1 | |
| HMDB01169_2 | 4-Aminophenol - multi-tags | 109.0528 | 288.5847 | 25.40 | 3.39 | 22.5 | |
| HMDB29306 | 4-Ethylphenol | 122.0732 | 356.1315 | 25.84 | 1.01 | 13.9 | |
| HMDB00500 | 4-Hydroxybenzoic acid | 138.0317 | 372.0900 | 17.75 | 2.30 | 4.4 | |
| HMDB01232 | 4-Nitrophenol | 139.0269 | 373.0853 | 23.61 | 1.69 | 8.9 | |
| HMDB01149 | 5-Aminolevulinic acid | 131.0582 | 365.1166 | 7.88 | 3.04 | 1.0 | |
| HMDB03355 | 5-Aminopentanoic acid | 117.0790 | 351.1373 | 9.10 | 1.28 | 9.8 | |
| HMDB00763 | 5-Hydroxyindoleacetic acid | 191.0582 | 425.1166 | 15.38 | 0.39 | 8.3 | |
| HMDB00450 | 5-Hydroxylysine | 162.1004 | 315.1085 | 13.85 | 0.62 | 12.4 | |
| HMDB00469 | 5-Hydroxymethyluracil | 142.0378 | 376.0962 | 9.56 | 1.14 | 26.8 | |
| HMDB01859 | Acetaminophen | 151.0633 | 385.1216 | 16.57 | 2.58 | 5.5 | |
| HMDB00050 | Adenosine | 267.0968 | 501.1551 | 4.42 | 0.20 | 9.4 | |
| HMDB00510 | Aminoadipic acid | 161.0688 | 395.1271 | 6.25 | 1.97 | 1.0 | |
| HMDB03012 | Aniline | 93.0578 | 327.1162 | 17.72 | 0.73 | 17.2 | |
| HMDB00056 | Beta-Alanine | 89.0477 | 323.1060 | 7.49 | 0.82 | 1.7 | |
| HMDB02322 | Cadaverine | 102.1157 | 285.1162 | 22.62 | 1.13 | 11.6 | |
| HMDB00904 | Citrulline | 175.0957 | 409.1540 | 3.79 | 1.46 | 17.4 | |
| HMDB02199 | Desaminotyrosine | 166.0630 | 400.1213 | 18.11 | 0.28 | 2.1 | Only detected in serum |
| HMDB00070 | D-Pipecolic acid | 129.0790 | 363.1373 | 13.45 | 2.12 | 2.5 | |
| HMDB00149 | Ethanolamine | 61.0528 | 295.1111 | 6.44 | 1.93 | 8.8 | |
| HMDB00112 | Gamma-Aminobutyric acid | 103.0633 | 337.1216 | 7.94 | 0.43 | 7.2 | |
| HMDB00152 | Gentisic acid | 154.0266 | 388.0849 | 17.43 | 1.44 | 12.3 | |
| HMDB00152_2 | Gentisic acid - multi-tags | 154.0266 | 311.0716 | 24.78 | 3.06 | 5.4 | |
| HMDB00131 | Glycerol | 92.0473 | 280.0820 | 22.91 | 0.71 | 0.5 | |
| HMDB00123 | Glycine | 75.0320 | 309.0903 | 6.95 | 1.72 | 4.5 | |
| HMDB00721 | Glycylproline | 172.0848 | 406.1431 | 7.43 | 0.69 | 1.3 | |
| HMDB01398 | Guaiacol | 124.0524 | 358.1107 | 22.76 | 1.43 | 11.5 | |
| HMDB01842 | Guanidine | 59.0483 | 293.1067 | 3.17 | 3.77 | 10.9 | Only detected in CSF |
| HMDB00133 | Guanosine | 283.0917 | 517.1500 | 2.49 | 1.20 | 5.4 | |
| HMDB00745 | Homocarnosine | 240.1222 | 354.1194 | 17.41 | 0.69 | 11.6 | Only detected in CSF |
| HMDB00130 | Homogentisic acid | 168.0423 | 318.0794 | 24.83 | 0.15 | 0.5 | Only detected in serum |
| HMDB00670 | Homo-L-arginine | 188.1273 | 422.1856 | 3.26 | 0.19 | 5.0 | |
| HMDB00118 | Homovanillic acid | 182.0579 | 416.1162 | 16.86 | 1.52 | 13.2 | |
| HMDB00755 | Hydroxyphenyllactici acid | 182.0579 | 416.1162 | 14.74 | 0.53 | 11.2 | |
| HMDB00157_3 | Hypoxanthine - Isomer | 136.0385 | 370.0968 | 10.00 | 1.52 | 6.5 | |
| HMDB00157_2 | Hypoxanthine - multi-tags | 136.0385 | 370.0968 | 8.98 | 1.56 | 0.2 | |
| HMDB00157 | Hypoxanthine + H2O | 136.0385 | 388.1098 | 2.54 | 3.94 | 3.5 | |
| HMDB02024 | Imidazoleacetic acid | 126.0429 | 360.1012 | 11.28 | 1.64 | 3.4 | |
| HMDB03320 | Indole-3-carboxylic acid | 161.0477 | 395.1060 | 19.30 | 0.76 | 3.6 | |
| HMDB00734 | Indoleacrylic acid | 187.0633 | 421.1216 | 21.02 | 2.27 | 21.3 | Only detected in serum |
| HMDB60003 | Isovanillic acid | 168.0423 | 402.1006 | 15.64 | 2.98 | 11.9 | |
| HMDB00704 | Isoxanthopterin | 179.0443 | 413.1026 | 9.77 | 0.62 | 1.1 | |
| HMDB00190 | Lactic acid | 90.0317 | 324.0900 | 11.96 | 1.54 | 0.9 | |
| HMDB00161 | L-Alanine | 89.0477 | 323.1060 | 7.90 | 1.54 | 3.3 | |
| HMDB00452 | L-Alpha-aminobutyric acid | 103.0633 | 337.1216 | 9.48 | 2.61 | 6.5 | |
| HMDB00517 | L-Arginine | 174.1117 | 408.1700 | 2.70 | 0.54 | 5.6 | |
| HMDB00168 | L-Asparagine | 132.0535 | 366.1118 | 3.25 | 3.34 | 5.6 | |
| HMDB00191 | L-Aspartic Acid | 133.0375 | 367.0958 | 5.45 | 3.42 | 1.3 | |
| HMDB00706 | L-Aspartyl-L-phenylalanine | 280.1059 | 514.1642 | 10.78 | 1.43 | 28.9 | |
| HMDB00099_2 | L-Cystathionine - Isomer | 222.0674 | 345.0920 | 13.87 | 0.10 | 0.3 | |
| HMDB00192 | L-Cystine | 240.0238 | 354.0702 | 14.17 | 2.38 | 6.6 | |
| HMDB00148 | L-Glutamic Acid | 147.0532 | 381.1115 | 5.32 | 3.97 | 2.5 | |
| HMDB00148_2 | L-Glutamic Acid - H2O | 147.0532 | 363.1009 | 9.71 | 3.01 | 0.5 | |
| HMDB00641 | L-Glutamine | 146.0691 | 380.1275 | 3.72 | 2.75 | 3.6 | |
| HMDB00177 | L-Histidine | 155.0695 | 389.1278 | 18.20 | 2.48 | 0.4 | |
| HMDB00719 | L-Homoserine | 119.0582 | 353.1166 | 4.51 | 2.30 | 8.2 | |
| HMDB00719_2 | L-Homoserine - H2O | 119.0582 | 335.1060 | 9.71 | 0.90 | 12.7 | |
| HMDB00172 | L-Isoleucine | 131.0946 | 365.1529 | 13.25 | 2.78 | 0.3 | |
| HMDB00687 | L-leucine | 131.0946 | 365.1529 | 13.54 | 2.53 | 0.0 | |
| HMDB00182 | L-Lysine | 146.1055 | 307.1111 | 17.50 | 0.72 | 5.3 | |
| HMDB00696 | L-Methionine | 149.0510 | 383.1094 | 11.26 | 2.63 | 9.5 | |
| HMDB00159 | L-Phenylalanine | 165.0790 | 399.1373 | 13.06 | 2.01 | 8.1 | |
| HMDB00162 | L-Proline | 115.0633 | 349.1216 | 10.54 | 2.45 | 8.1 | |
| HMDB00187 | L-Serine | 105.0426 | 339.1009 | 4.71 | 3.04 | 1.1 | |
| HMDB00167 | L-Threonine | 119.0582 | 353.1166 | 6.12 | 2.83 | 1.6 | |
| HMDB00929 | L-Tryptophan | 204.0899 | 438.1482 | 11.65 | 1.29 | 0.0 | |
| HMDB00158 | L-Tyrosine | 181.0739 | 324.5953 | 22.67 | 0.39 | 0.9 | |
| HMDB00883 | L-Valine | 117.0790 | 351.1373 | 11.19 | 2.73 | 9.8 | |

| | | | | | | | |
|---|---|---|---|---|---|---|---|
| HMDB02048 | m-Cresol | 108.0575 | 342.1158 | 24.58 | 2.69 | 8.3 | |
| HMDB02005 | Methionine Sulfoxide | 165.0460 | 399.1043 | 3.94 | 2.23 | 7.0 | |
| HMDB02005_2 | Methionine Sulfoxide - Isomer | 165.0460 | 399.1043 | 4.38 | 1.12 | 9.2 | |
| HMDB02108 | Methylcysteine | 135.0354 | 369.0937 | 9.80 | 1.43 | 11.4 | |
| HMDB01522 | Methylguanidine | 73.0640 | 307.1223 | 4.34 | 0.56 | 10.1 | |
| HMDB02172 | N1,N12-Diacetylspermine | 286.2369 | 377.1768 | 14.82 | 1.20 | 0.9 | |
| HMDB02064 | N-Acetylputrescine | 130.1106 | 364.1689 | 7.37 | 1.74 | 9.5 | |
| HMDB00446 | N-Alpha-acetyllysine | 188.1161 | 422.1744 | 7.23 | 0.21 | 9.6 | |
| HMDB02141 | N-Methyl-a-aminoisobutyric acid | 117.0790 | 351.1373 | 13.97 | 3.88 | 9.1 | |
| HMDB02393 | N-methyl-D-aspartic acid | 147.0532 | 381.1115 | 8.03 | 0.18 | 13.8 | |
| HMDB02055 | o-Cresol | 108.0575 | 342.1158 | 24.80 | 1.37 | 12.3 | |
| HMDB00224 | O-Phosphoethanolamine | 141.0191 | 375.0774 | 2.14 | 1.90 | 15.0 | |
| HMDB00214 | Ornithine | 132.0899 | 300.1033 | 16.64 | 2.28 | 4.4 | |
| HMDB03337 | Oxidized glutathione | 612.1520 | 540.1343 | 8.16 | 3.05 | 10.4 | |
| HMDB01392 | p-Aminobenzoic acid | 137.0477 | 371.1060 | 11.63 | 4.31 | 5.8 | |
| HMDB00210 | Pantothenic acid | 219.1107 | 453.1690 | 8.53 | 1.25 | 6.2 | |
| HMDB00228 | Phenol | 94.0419 | 328.1002 | 23.51 | 0.73 | 19.9 | |
| HMDB13302 | Phenylalanylphenylalanine | 312.1474 | 546.2057 | 16.59 | 0.50 | 5.3 | Only detected in serum |
| 316 | Phenyl-Leucine | 278.1631 | 512.2214 | 15.91 | 3.38 | 7.9 | Only detected in serum |
| HMDB00020 | p-Hydroxyphenylacetic acid | 152.0473 | 386.1057 | 17.26 | 1.65 | 14.1 | |
| HMDB01545 | Pyridoxal | 167.0582 | 401.1166 | 12.38 | 2.42 | 10.4 | |
| HMDB00884_3 | Ribothymidine - H2O | 258.0852 | 474.1329 | 9.68 | 1.53 | 2.9 | |
| HMDB00884_2 | Ribothymidine - Isomer | 258.0852 | 492.1435 | 8.70 | 0.51 | 5.7 | |
| HMDB00279 | Saccharopine | 276.1321 | 510.1905 | 2.60 | 2.20 | 1.2 | |
| HMDB00279_2 | Saccharopine - H2O | 276.1321 | 492.1799 | 5.99 | 1.36 | 2.2 | |
| HMDB01895 | Salicylic acid | 138.0317 | 372.0900 | 15.86 | 1.39 | 6.1 | |
| HMDB00840 | Salicyluric acid | 195.0532 | 429.1115 | 11.29 | 0.08 | 1.3 | Only detected in serum |
| HMDB00271 | Sarcosine | 89.0477 | 323.1060 | 9.65 | 0.93 | 3.7 | |
| HMDB00259 | Serotonin | 176.0950 | 322.1058 | 24.85 | 3.97 | 12.1 | Only detected in serum |
| HMDB03334 | Symmetric dimethylarginine | 202.1430 | 436.2013 | 3.52 | 0.41 | 7.4 | |
| HMDB00251 | Taurine | 125.0147 | 359.0730 | 2.58 | 2.31 | 0.9 | |
| HMDB01918 | Thyroxine | 776.6867 | 622.4017 | 27.68 | 0.11 | 0.4 | Only detected in serum |
| HMDB00725 | Trans-4-Hydroxyl-L-Proline | 131.0582 | 365.1166 | 5.44 | 3.07 | 2.3 | |
| HMDB00300 | Uracil | 112.0273 | 346.0856 | 11.81 | 1.30 | 15.6 | |
| HMDB00296 | Uridine | 244.0695 | 478.1279 | 8.13 | 1.69 | 1.1 | |
| HMDB00296_2 | Uridine - H2O | 244.0695 | 460.1173 | 8.89 | 1.06 | 2.3 | |
| HMDB00301 | Urocanic acid | 138.0429 | 372.1012 | 13.70 | 0.41 | 0.2 | |
| HMDB00484 | Vanillic acid | 168.0423 | 402.1006 | 17.79 | 0.20 | 20.4 | |
| HMDB00291 | Vanillylmandelic acid | 198.0528 | 432.1111 | 13.02 | 1.14 | 1.5 | |
| HMDB00292 | Xanthine | 152.0334 | 386.0917 | 9.33 | 1.68 | 7.9 | |

Supplemental Table T2. Summary of pathway analysis results.

| Pathway Name from CSF Data | Total | Hits | Raw *p* | -log(*p*) | Impact |
|---|---|---|---|---|---|
| Pyrimidine metabolism | 60 | 1 | 1.10E-07 | 16.02 | 0.02 |
| Aminoacyl-tRNA biosynthesis | 75 | 5 | 9.39E-06 | 11.58 | 0.06 |
| Arginine and proline metabolism | 77 | 6 | 1.87E-05 | 10.89 | 0.27 |
| Histidine metabolism | 44 | 1 | 9.40E-05 | 9.27 | 0.05 |
| Lysine biosynthesis | 32 | 1 | 2.39E-04 | 8.34 | 0.10 |
| Biotin metabolism | 11 | 1 | 2.39E-04 | 8.34 | 0.00 |
| D-Arginine and D-ornithine metabolism | 8 | 1 | 2.80E-04 | 8.18 | 0.00 |
| Lysine degradation | 47 | 2 | 4.99E-04 | 7.60 | 0.16 |
| Glycine, serine and threonine metabolism | 48 | 2 | 7.29E-04 | 7.22 | 0.10 |
| Valine, leucine and isoleucine biosynthesis | 27 | 2 | 1.19E-03 | 6.73 | 0.01 |
| Glycerophospholipid metabolism | 39 | 1 | 1.90E-03 | 6.26 | 0.06 |
| Nitrogen metabolism | 39 | 2 | 2.25E-03 | 6.10 | 0.00 |
| Porphyrin and chlorophyll metabolism | 104 | 1 | 2.93E-03 | 5.83 | 0.00 |
| Pyruvate metabolism | 32 | 1 | 3.90E-03 | 5.55 | 0.14 |
| Glycolysis or Gluconeogenesis | 31 | 1 | 3.90E-03 | 5.55 | 0.00 |
| Propanoate metabolism | 35 | 1 | 3.90E-03 | 5.55 | 0.00 |
| Tryptophan metabolism | 79 | 2 | 5.53E-03 | 5.20 | 0.07 |
| Phenylalanine, tyrosine and tryptophan biosynthesis | 27 | 1 | 1.02E-02 | 4.58 | 0.07 |
| Valine, leucine and isoleucine degradation | 40 | 1 | 1.31E-02 | 4.33 | 0.00 |
| Cysteine and methionine metabolism | 56 | 1 | 1.80E-02 | 4.02 | 0.13 |
| Glycerolipid metabolism | 32 | 1 | 2.36E-02 | 3.75 | 0.19 |
| Galactose metabolism | 41 | 1 | 2.36E-02 | 3.75 | 0.00 |
| Alanine, aspartate and glutamate metabolism | 24 | 1 | 5.05E-02 | 2.99 | 0.10 |
| Butanoate metabolism | 40 | 1 | 5.05E-02 | 2.99 | 0.01 |
| beta-Alanine metabolism | 28 | 1 | 5.05E-02 | 2.99 | 0.00 |
| Glutathione metabolism | 38 | 2 | 8.27E-02 | 2.49 | 0.00 |
| **Pathway Name from Serum Data** | **Total** | **Hits** | **Raw *p*** | **-log(*p*)** | **Impact** |
| Pyrimidine metabolism | 60 | 1 | 4.79E-07 | 14.55 | 0.02 |
| Phenylalanine metabolism | 45 | 1 | 1.15E-05 | 11.37 | 0.12 |
| Phenylalanine, tyrosine and tryptophan biosynthesis | 27 | 1 | 1.15E-05 | 11.37 | 6.2E-04 |
| Nitrogen metabolism | 39 | 1 | 1.15E-05 | 11.37 | 0.00 |
| Aminoacyl-tRNA biosynthesis | 75 | 1 | 1.15E-05 | 11.37 | 0.00 |

| | | | | | |
|---|---|---|---|---|---|
| **Arginine and proline metabolism** | 77 | 3 | 4.80E-05 | 9.95 | 0.08 |
| **Glycine, serine and threonine metabolism** | 48 | 1 | 5.48E-03 | 5.21 | 0.05 |
| **Tyrosine metabolism** | 76 | 2 | 6.43E-03 | 5.05 | 1.9E-04 |
| **Ubiquinone and other terpenoid-quinone biosynthesis** | 36 | 1 | 1.05E-02 | 4.56 | 0.04 |

Supplemental Table T3. Summary of ROC curve analysis of 7 discriminant metabolites.

| Metabolite<br>A vs. B | AUC<br>(95% CIs) | Optimal threshold | Sensitivity | Specificity |
|---|---|---|---|---|
| Citrulline | 0.608 (0.367-0.850) | 1.06 | 0.70 | 0.50 |
| N-Acetylputrescine | 0.658 (0.426-0.891) | 1.24 | 0.70 | 0.58 |
| N-Methyl-D-aspartic acid | 0.729 (0.508-950) | 1.90 | 0.70 | 0.67 |
| N1,N12-Diacetylspermine | 0.858 (0.705-1.000) | 0.68 | 0.80 | 0.75 |
| Lactic acid | 0.817 (0.635-0.999) | 0.92 | 0.80 | 0.67 |
| Glycerol | 0.833 (0.662-1.000) | 0.88 | 0.80 | 0.75 |
| 5-Hydroxylysine | 0.675 (0.425-0.925) | 1.66 | 0.90 | 0.67 |
| **Metabolite<br>A vs. C** | **AUC<br>(95% CIs)** | **Optimal threshold** | **Sensitivity** | **Specificity** |
| Citrulline | 0.914 (0.777-1.000) | 0.74 | 0.90 | 0.86 |
| N-Acetylputrescine | 0.857 (0.676-1.000) | 1.01 | 0.90 | 0.71 |
| N-Methyl-D-aspartic acid | 0.879 (0.703-1.000) | 1.64 | 0.80 | 0.86 |
| N1,N12-Diacetylspermine | 0.943 (0.824-1.000) | 0.57 | 1.00 | 0.86 |
| Lactic acid | 0.929 (0.807-1.000) | 0.91 | 0.80 | 0.86 |
| Glycerol | 0.900 (0.753-1.000) | 0.86 | 0.80 | 0.86 |
| 5-Hydroxylysine | 0.871 (0.703-1.000) | 1.51 | 0.80 | 0.86 |
| **Metabolite<br>B vs. C** | **AUC<br>(95% CIs)** | **Optimal threshold** | **Sensitivity** | **Specificity** |
| Citrulline | 0.786 (0.576-0.996) | 0.76 | 0.75 | 0.86 |
| N-Acetylputrescine | 0.690 (0.447-0.934) | 1.04 | 0.67 | 0.71 |
| N-Methyl-D-aspartic acid | 0.744 (0.516-972) | 1.35 | 0.67 | 0.71 |
| N1,N12-Diacetylspermine | 0.738 (0.489-0.987) | 0.31 | 0.92 | 0.57 |
| Lactic acid | 0.560 (0.292-0.827) | 0.72 | 0.58 | 0.57 |
| Glycerol | 0.631 (0.354-0.908) | 0.54 | 0.75 | 0.57 |
| 5-Hydroxylysine | 0.560 (0.276-0.843) | 1.91 | 0.50 | 0.71 |

Supplemental Table T4. Summary of logistic regression models for predicting ASIA impairment grades using identified metabolites.*

| | | Predicted ASIA grade | | | |
|---|---|---|---|---|---|
| | | A | B | C | Total |
| **Observed ASIA grade** | A | 8 | 1 | 1 | 10 |
| | B | 2 | 8 | 2 | 12 |
| | C | 0 | 2 | 5 | 7 |
| | Total | 10 | 11 | 8 | 29 |

*The workflow and procedure used for generating the results in the table are shown below.

In the first step of analysis, logistic regression was conducted on the metabolites for evaluating binary classification of the injury severities A and non-A, and then a greedy stepwise backward selection was carried out on the samples of the data using the identified metabolites measurable in CSF and serum as well as their classes "A" and "non-A". The selection procedure produced a ranking of the importance of metabolites as a predictor of the injury severity in terms of A and non-A. The selected metabolites were citrulline, glycerol and N-methyl-D-aspartic acid in the first step of analysis.

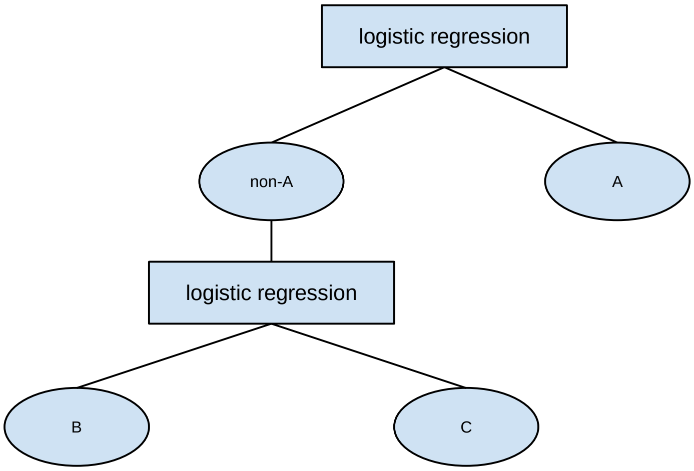


In the second step of analysis, logistic regression and greedy stepwise backward selection were conducted on the metabolites with patients of assigned grades "non-A" from the previous step. The selected metabolites were citrulline and glycerol in the second step. Logistic regression was conducted on the selected metabolites as a predictor for injury severity of B and C.

Below show the equations for generating the above table.

**First step:**

The logistic regression model with the selected metabolites as input variables x = (Citrulline, N-methyl-D-aspartic-acid, glycerol) learns their coefficients w = (2.5578, 1.9759, 5.916) and intercept 12.6459 and then calculates the class membership probability for A and NonA patients in the data set.

The hyperplane of all points *x* satisfying the equation *x' * w + intercept*=0 forms the *decision boundary* between the two classes, which is given by

x’w + intercept = 2.5578 * Citrulline + 1.9759 * ( N-methyl-D-aspartic acid) + 5.916 * glycerol – 12.6459

Patients with 1 / (1 + exp(-x’w + intercept)) above the threshold of 0.5 were classified as A.

**Second Step:**

Likewise, in the second step, the logistic regression model with the selected metabolites as input variables x = (citrulline, glycerol) learns their coefficients w = (4.0015,3.4475) and the intercept - 5.2712 and then calculates the class membership probability for B and C patients in the data set.

P(B | x, w, intercept) = 1 / (1 + exp(-x’w + intercept));

P(C | x, w, intercept) = 1 - P(B | x, w, intercept).

where weighted_sum = x’w + intercept = 4.0015 * Citrulline + 3.4475 * glycerol - 5.2712

Patients with 1 / (1 + exp(-x’w + intercept)) above the threshold of 0.5 were classified as B.

Supplemental Table T5. Summary of logistic regression models for predicting ASIA impairment grades using putative metabolites in CSF.*

| | | Predicted ASIA grade | | | |
|---|---|---|---|---|---|
| | | A | B | C | Total |
| **Observed ASIA grade** | A | 9 | 0 | 1 | 10 |
| | B | 0 | 11 | 1 | 12 |
| | C | 0 | 0 | 7 | 7 |
| | Total | 9 | 11 | 9 | 29 |

*The number ID of putative metabolites used in the first step were C2717, C2094, C296, C114, C162 and C315, and in the second step were C1147, C3936 and C296.

The same two-step procedure as noted in Supplemental Table T3 was conducted on the putative metabolites in CSF for all patients.

Logistic Regression Equation for the above table:

**First Step:**

The logistic regression model with the top six selected metabolites as input variables x = (C2717, C2904, C296, C114, C162, C315) learns their coefficients w = ( -83.435,15.3184,16.0929, 20.1932, -13.9344, 7.1853) and intercept -31.7988 and then calculates the class membership probability for A and NonA patients in the data set.

weighted_sum = x’ w + intercept= -83.435 * C2717 + 15.3184 * C2904 + 16.0929 * C296 + 20.1932 * C114 + ( -13.9344) * C162 + 7.1853 * C315 -31.7988

Patients with 1/(1 + exp(-weighted_sum)) above the threshold of 0.5 were classified as A; otherwise, non-A

**Second Step:**

In the second step, the logistic regression model with the selected metabolites as input variables x = (C1147, C3936, C296) learns their coefficients w = ( 96.9603,269.3487,-85.4335) and the intercept -204.4642 and then calculates the class membership probability for B and C patients in the data set.

weighted_sum = x’ w + intercept = 96.9603* C1147 + 269.3487 * C3936 -85.4335 * C296 - 204.4642

Patients with 1/(1 + exp(-weighted_sum)) above 0.5 were classified as B; otherwise C.

Supplemental Table T6. Summary of logistic regression models for predicting ASIA impairment grades using putative metabolites in serum.*

| | | Predicted ASIA grade | | | |
|---|---|---|---|---|---|
| | | A | B | C | Total |
| **Observed ASIA grade** | A | 8 | 2 | 0 | 10 |
| | B | 0 | 12 | 0 | 12 |
| | C | 1 | 1 | 5 | 7 |
| | Total | 9 | 15 | 5 | 29 |

*The number ID of putative metabolites used in the first step were S15780, S2496 and S1053, and in the second step was S13214.

The same two-step procedure as noted in Supplemental Table T3 was conducted on the putative metabolites in serum for all patients.

Logistic Regression Equation for the above table:

**First Step:**

The logistic regression model with the top six selected metabolites as input variables x = (S15780, S2496, S1053) learns their coefficients w = (5.316, 1.7525, 1.3822) and intercept – 13.238 and then calculates the class membership probability for A and NonA patients in the data set.

weighted_sum = x'w + intercept = 5.316 * S15780 + 1.7525 * S2496 + 1.3822 * S1053 – 13.238

Patients with 1/(1 + exp(-weighted_sum)) above 0.5 were classified as class A; otherwise, non-A.

**Second Step:**

In the second step, the logistic regression model with the selected metabolites as input variables x = (S13214) learns their coefficients w = (-13.8837) and the intercept 24.8233 and then calculates the class membership probability for B and C patients in the data set.

weighted_sum = x w + intercept = -13.8837 * S13214 + 24.8233

Patients with 1/(1 + exp(-weighted_sum)) above the threshold of 0.5 were predicted as B; otherwise C.

Supplemental Table T7. Summary of logistic regression models for predicting ASIA impairment grades using putative metabolites in serum/CSF.*

| | Predicted ASIA grade | | | | |
|---|---|---|---|---|---|
| | | A | B | C | Total |
| **Observed ASIA grade** | A | 10 | 0 | 0 | 10 |
| | B | 1 | 11 | 0 | 12 |
| | C | 0 | 1 | 6 | 7 |
| | Total | 11 | 12 | 6 | 29 |

*The number ID of putative metabolites used in the first step were S15780, C4688, S2496, S143 and S984, and in the second step were S13214 and S984.

The same two-step procedure as noted in Supplemental Table T3 was conducted on the putative metabolites in both serum and CSF for all patients.

Logistic Regression Equation for the above table:

**First Step:**

The logistic regression model with the top six selected metabolites as input variables x = (S15780, C4688, S2496, S143, S984) learns their coefficients w = (61.739, 54.2275,23.431, 16.09, 62.5507) and intercept – 273.0694 and then calculates the class membership probability for A and NonA patients in the data set.

weighted_sum = x' w + intercept = 61.739 * S15780 + 54.2275 * C4688 + 23.431 * S2496 + 16.09 * S143 + 62.5507 * S984 – 273.0694

Patients with 1/(1 + exp(-weighted_sum)) above the threshold of 0.5 were predicted as A; otherwise Non-A

**Second Step:**

In the second step, the logistic regression model with the selected metabolites as input variables x = (S13214, S984) learns their coefficients w = (-326.8552, - 211.1922) and the intercept 758.648 and then calculates the class membership probability for B and C patients in the data set.

weighted_sum = x'w + intercept = -326.8552 * S13214 - 211.1922 * S984 + 758.648

Patients with 1/(1 + exp(-weighted_sum)) above the threshold of 0.5 were classified as B; otherwise, C.

Supplemental Table T8. List of top selected metabolites for building the prediction models.

| Number ID | RT | Dansylated mass | Neutral mass | MCID with 0 reaction or HMDB* | # of matches in MCID with 1 reaction |
|---|---|---|---|---|---|
| **C2717** | 11.46 | 409.1072 | 175.0489 | 2-Amino-3-oxoadipate | / |
| **C2094** | 9.78 | 405.1174 | 342.1182 | Disaccharide isomers such as sucrose | / |
| **C296** | 3.25 | 355.1337 | 121.0754 | / | 21 |
| **C114** | 2.46 | 380.0813 | 146.023 | / | 29 |
| **C162** | 2.60 | 320.0443 | 85.98594 | / | / |
| **C315** | 3.34 | 558.1657 | 324.1074 | / | 31 |
| **C1147** | 7.30 | 337.1215 | 103.0632 | b-aminoisobutyric acid<br>N-Ethylglycine | / |
| **C3936** | 14.49 | 518.1699 | 284.1115 | / | 18 |
| **C4688** | 15.61 | 308.0939 | 74.0356 | 3-Hydroxypropanal<br>Lactaldehyde<br>Hydroxyacetone | / |
| **S15780** | 30.28 | 606.3466 | 372.2882 | / | 15 |
| **S2496** | 10.82 | 405.1484 | 171.0901 | / | 30 |
| **S1053** | 7.10 | 414.1203 | 180.0620 | Monosaccharide isomers such as glucose | / |
| **S13214** | 26.93 | 369.1021 | 270.0875 | / | 16 |
| **S143** | 2.54 | 295.114 | 61.0531 | / | 15 |
| **S984** | 6.92 | 707.1188 | 240.0095 | / | / |

*Accurate masses were first searched against the MyCompoundID database with zero reaction, which contain only endogenous metabolites. If there was no match, the accurate masses were then searched against the HMDB database. If there was still no match, MyCompoundID database with one reaction was used and the number of matches was listed in this table. Only metabolites with plausible structures (i.e., can be dansyl-labeled) were listed. Search criteria were mass error < 5 ppm. Some matches were excluded from the list because the retention time did not match with the dansyl standard library.
